# *In vivo* anti-ulceration effect of *Pancratium maritimum* extract against ethanol-induced rats via NLRP3 inflammasome and HMGB1/TLR4/MYD88/NF-κβ signaling pathways and its extract metabolite profile

**DOI:** 10.1371/journal.pone.0321018

**Published:** 2025-04-16

**Authors:** Rehab F. Taher, Eman M. Abd El ghany, Zeinab A. El-Gendy, Mai M. Elghonemy, Heba A. Hassan, Gehad A. Abdel Jaleel, Azza Hassan, Tushar C. Sarker, Ahmed M. Abd-ElGawad, Mohamed A. Farag, Abdelsamed I. Elshamy

**Affiliations:** 1 Department of Natural Compounds Chemistry, National Research Centre, Dokki, Giza, Egypt; 2 Pharmacognosy Department, Faculty of Pharmacy, Cairo University, Cairo, Egypt; 3 Department of Pharmacology, Medical Research and Clinical Studies Institute, National Research Centre, Dokki, Giza, Egypt; 4 Therapeutic Chemistry Department, National Research Centre, Dokki, Giza, Egypt; 5 Department of Pathology, Faculty of Veterinary Medicine, Cairo University, Cairo, Egypt; 6 Texs A&M AgriLife Research Center, Overton, Texas, United States of America; 7 Plant Production Department, College of Food & Agriculture Sciences, King Saud University, Riyadh, Saudi Arabia; 8 Healthcare Department, Saxony Egypt University (SEU), Badr City, Egypt; Bowen University, Nigeria

## Abstract

**Background:**

Gastric ulcer is a multifaceted ailment of multiple causes and is chronic warranting the discovery of remedies to alleviate its symptoms and severity. *Pancratium maritimum* L. is recognized for its several health benefits, although its potential against gastric ulcers has yet to be reported.

**Methods and findings:**

This study reports on the effects of *P. maritimum* L. whole plant (PM-EtOH) ethanol extract at a dose of 25, 50, and 100 mg/kg body weight orally for managing ethanol-induced peptic ulcer in rats. The anti-ulceration capacity of PM-EtOH was determined against ethanol (EtOH)-induced rats *via* biochemical, histological, immunohistochemical, and western blotting assays. The profiling of the bioactive metabolites in *P. maritimum* extract was based on Ultra-high-performance liquid chromatography coupled with electrospray ionization quadrupole time-of-flight mass spectrometry (UHPLC-ESI-qTOF-MS/MS) analysis. Following PM-EtOH treated group, the gastric glutathione (GSH) level dropped in the ulcer group receiving ethanol was restored to normal levels. Additionally, following PM-EtOH, elevated malondialdehyde (MDA) content in the stomach tissues diminished. PM-EtOH treated group displayed recovery and comparable morphology compared with normal group, concurrent with lower levels of Tumor Necrosis Factor α (TNF-α), MyD88, and NLRP3, along with low expression of Nuclear Factor kappa *β* (NF-к*β*) and high-mobility group box protein 1 (HMGB1) proteins. Immune-histochemicals of caspase-3 and toll-like receptors-4 (TLR-4) showed their normalization. These findings imply that PM-EtOH exerts a protective effect on rat stomach damage that has yet to be further tested in clinical trials for treatment of stomach ulcers. Phytochemical profiling of PM-EtOH *via* UHPLC-ESI-qTOF-MS/MS led to the identification of 84 metabolites belonging to amino acids, organic acids, phenolic acids, alkaloids, flavonoids, and fatty acids to likely mediate for the observed effects.

**Conclusions:**

These outcomes provided evidence for the potential of PM-EtOH in gastric ulcers management.

## 1. Introduction

Gastric ulcer is a long-term disorder that develops damage to the gastric mucosa [1]. Disparity between protective elements as mucus and bicarbonate production, epithelial cell barrier integrity, and sufficient blood flow and aggressive variables like high stomach acid production, pepsin activity, the presence of *Helicobacter pylori*, or exogenous substances such as ethanol,“ all factors affecting the luminal surface of epithelial cells, as well as subsequent injury in the gastro-duodenal mucosa caused by immunologic or environmental factors, are aspects that engender peptic ulcer [2]. Exacerbating variables comprise *Helicobacter pylori*, HCl, pepsin, non-steroidal anti-inflammatory medicine (NSAIDs), bile acids, ischemia, hypoxia, smoking, and alcohol [3]. Contrarily, the protective factors include growth factors, glutathione (GSH), bicarbonate, nitric oxide (NO), prostaglandins (PGs), as well as enzymes acting as antioxidants or peptides [4].

The term “peptic ulcer” refers to epithelial sores that penetrate muscularis mucosae layer, creating a hollow encircled by both acute and chronic inflammation [5]. Accessible pharmacological regimes that are available to promote mucosal damage healing and prevent peptic ulcers include PG analogs, proton pump inhibitors, histamine H2 receptor antagonists (H2RAs), antibiotics, and cytoprotective medications [6]. Nevertheless, all current regimens have side effects i.e., gastric disturbance comprising vomiting, nausea, diarrhea, constipation, and cardiovascular effects [7]. Other side effects include impotence, hypoacidity, gynecomastia, osteoporotic bone fractures, and hypergastrinemia [6]. Hence, there is a need to identify medications that are less risky, and affordable [8].

Inflammation is caused by exposure to various stimuli, such as ROS, which oxidize cellular proteins and lipids, leading to the disruption of the intestinal tract barrier and increasing gut permeability [9]. Nuclear protein HMGB1 is a chromatin-binding factor that regulates the transcription of many nuclear genes and stabilizes nucleosomes [10]. Extracellular HMGB1, on the other hand, is a strong cytokine that is proinflammatory and released by monocytes and macrophages or leaks during cell necrosis. HMGB1 binds to many receptors, including Toll-like receptor 4 (TLR-4) [11]. After attaching itself to its receptors, HMGB1 causes inflammation *via* cell differentiation as TLR-4 produces MyD88, a component of the NF-к*β* pathway, in response to ligand recognition [12]. Inhibitor of kappa B (IкB) protein in the cytoplasm releases Nuclear factor kappa-light-chain-enhancer of activated B cells/p65 subunit (NF-к*β*/p65), which then translocate to the nucleus as it activates the transcription of additional pro-inflammatory cytokines including TNF-α [13] and Leucine-rich repeat-containing protein 3 (NLRP3) [14]. NLRP3 processes TNF-α precursor, facilitating its development. TNF-α inhibits stomach microcirculation delaying ulcerated mucosal healing [15]. Therefore, inhibition of the activation of NLRP3 inflammasome and HMGB1/TLR4/MYD88/NF-к*β* signaling pathway can be a therapeutic target for the treatment of inflammation.

Contemporary herbal medicine is increasingly emerging as a potential source of drug discovery [16]. *Pancratium maritimum* L. or (sea daffodil), Amaryllidaceae Family, is a perennial Mediterranean plant that is typical of sandy coastal environments [17, 18]. Previous papers have revealed its richness in chemical classes including alkaloids, flavonoids [19], phenolic acids [19, 20], chromones, acetophenones [19], phenylpropanoids [21], and alginates [22]. Taking into account its intriguing medical value, the plant was reported to exhibit several health-promoting activities, such as purgative, acaricidal, insecticidal, and antifungal activities for the leaf and bulb extracts [17]. The whole plant was utilized in folk medicine as an emetic, hypotensive, and for treatment of spleen inflammation [19]. Furthermore, the anticancer [21,23], antimicrobial, antiviral [24], radical scavenging [23], antinociceptive [25], amoebicidal [26], antimalarial [27], and acetylcholinesterase inhibiting [28] activities of extracts from several *P. maritimum* accessions in addition to identified compounds have been reported.

For the first time in the literature, the current investigation targeted to: (i) examine PM-EtOH’s anti-ulcerogenic ability against ethanol-induced ulceration in rate model; (ii) carry out a comprehensive metabolites profile analysis of PM-EtOH employing UHPLC-ESI-Orbitrap-MSUHPLC-ESI-Orbital Trap-MS; and (iii) study the mechanism of action linked to the metabolites profile through biochemical and histopathological assays.

## 2. Materials and methods

### 2.1. Plant collection and authentication

Throughout the flowering season at the end of April 2022, the entire plant of *Pancratium maritimum* was collected from the Egyptian eastern desert in Wadi Hagul (30°02′34.3″ N, 32°05′40.6″ E). Prof. Dr. Ahmed M. Abdel Gawad, a plant ecology professor authenticated the plant specimen. A voucher specimen (896-PM-2-ByH/22–00562) is deposited at the Herbarium of the Faculty of Science, Mansoura University in Egypt.

### 2.2. Preparation of the extract

The collected plant material was thoroughly cleaned to remove dirt or sand and placed into an open, shaded room that was perfectly dried at room temperature. Following drying, it was ground up into a powder with a sanitized plant grinder. The plant powder (700 g) was extracted for three days by maceration in 70% EtOH (3 L) at 25–28 °C. After filtration, the collected extract was thoroughly dried under low pressure to yield 28.7 g of sticky dark black residue. Dried extract that is devoid of EtOH was stored at 4 °C till further biological or chemical analysis.

### 2.3. Acute toxicity test

All biological studies were conducted in accordance with the Ethical Committee of the Medical Research Ethics Committee and Animal Care and Use Committee (ACUC), and National Research Center (approval number; 513012023). Six Wistar rats, males, were utilized to assess oral acute toxicity of PM-EtOH under investigation following the procedure with the starting dose of 2000 mg/kg b.w. of the extract mentioned in OCED guidelines. Six overnight fasted rats were administered 2000 mg/kg b.w. of PM-EtOH orally for seven days. The toxicity, behavioral alteration, any clinical signs of toxicity, and mortality were observed (OECD, 2000).

### 2.4. Gastroprotection assay

#### 2.4.1. Chemicals and drugs.

Acetonitrile, formic acid (≥ 95.0%, FA), water, and methanol (LC-MS grade) were purchased from Merck (Darmstadt, Germany). Ethanol (96%) was also purchased from Merck Millipore Burlington, Massachusetts, USA. Omeprazole was acquired from Sigma-Aldrich.

#### 2.4.2. Animals.

A total of 36 male mature Wistar albino rats, each weighing between 200 and 250 grams, were obtained from the animal house at the National Research Centre. Every animal was housed in metal cages with adequate ventilation, maintained at 22 °C with 12-h cycles of light and darkness. They were given regular rat meal pellets, which included an unrestricted supply of water along with 21% proteins, 3.48% fats, 3.71% raw fiber, and 1% multivitamins. The contents comprise limestone, hulled sunflower cake, soybean meal (44%), gluten of corn (60%), yellow maize, raw oil of soybean, methionine, a blend of minerals and vitamins along with an anti-fungicide. As well, water was constantly on hand for the experiment. The study’s methodologies and procedures were carried out in compliance with the Institutional Care and Use Committee (IACUC), National Institutes of Health’s regulations (NIH publication No. 85–23, modified 2011), the National Research Center in Egypt’s Ethics Committee (registration number 513012023) and the ARRIVE guidelines [29]. Throughout the whole duration of the experiment any necessary steps were carried out to eliminate the rats’ pain, suffering, and loss of weight.

#### 2.4.3. Experimental design.

In this study, rats were housed for 12 h a day, 12 h at night, with sufficient water and food to let them adjust to their surroundings. On the second day following adjusting, the rats were separated into 6 groups at random, of 6 rats per group. On the 7^th^ day, every group of rats was divided into the following categories, except for the initial group that was given 1 ml/kg of ethanol (96%) with gavage [30].

**First** group (normal): normal rats; **second** group (control): ulcer control rats; **third** group (standard group): Omeprazole 20mg/kg [14]; **fourth, fifth, and sixth** groups (experimental groups): received PM-EtOH at a dose of 25, 50, and 100 mg/kg by intra-gastric force-feeding for 7 successive days before ulcer induction. To recreate the stress conditions caused by the drug intra-gastric force-feeding in the third through sixth groups, the first and second groups were given 10 mg/kg body mass normal saline (0.0.9%).

All necessary precautions were implemented to prevent and/or reduce the pain and/or suffering by performing actions like humanely eliminating the suffering or reducing it. From the first day until the experiment endpoint, animals were checked out a minimum of once a day, and subsequently twice a day. A qualified laboratory animal technologist conducted the tests and evaluations. The respiratory rates and efforts of every animal observed varied from weak to normal. Behavior, postural shifts, and mobility were also observed. Every day till the end of the experiment, the body weight of each rate was tracked individually. Additionally, a Universal Interface Device (UID) reader was employed once or twice throughout the day for determining body temperature. Before the planned euthanasia at the time of fulfilling humane endpoints, the animals’ body temperatures were recorded using a non-contact electronic infrared thermometer (Lasergrip 774, Etekcity Inc., Anaheim, CA, USA) while they were being gently restrained by their scruffs.Upon completion of the experiment, all rats were humanely euthanized using an intraperitoneal injection of sodium phenobarbital (40–50 mg/kg). Euthanasia was carried out in line with certain behavioral signs of severe pain, such as hunched posture, reluctance to move, excessive licking of the abdomen, or guarding the abdominal area, particularly when the pain persisted despite the administration of analgesics (meloxicam 2mg/kg ip). Throughout the experiment, no animals died early or before they reached a humane endpoint. All animals stayed in good health during the research, and no unexpected deaths occurred. The rats were subsequently disposed of in compliance with the recommendations established out through the National Research Center’s Safety and Health Committee (NRC). Animal stomachs were rapidly removed, then parted along the larger curve, removing their contents in the process. The stomach tissue specimens were macroscopically examined to determine the stomach ulcer index after being gently cleaned with a cold saline solution buffered with phosphate to remove any blood clots. Between two filter papers, the stomach was dried and split into 3 parts; first part was utilized to prepare 10% homogenate *via* homogenization in ice-cold saline to evaluate oxidative stress markers and antioxidant characteristics, and then frozen at -20 °C. The remaining portion was maintained at -80 °C in preparation for a later Western blot examination. Ultimately, the third section was embedded in 10% formalin for histological examination.

#### 2.4.4. Macroscopical examination.

Using an X-10 magnifying lens, lesion counts were tallied on flattened stomach samples that were photographed. Calculations were used to determine the ulcer inhibition percentage (%I) and ulcer index (UI) in square mm^2^, slightly differently from the process pointed out by Takagi & Okabe [31]. Using a ruler, the surface of the wound was initially measured in this technique, and the degree of the ulcer was used to assess its severity (S1 Table).

#### 2.4.5. Histological analysis.

All animal groups’ stomach tissues were divided into pieces, which were then cut up and preserved in 10% buffered formalin. After fixation, the samples were washed with water, and thereafter encased in paraffin wax. Subsequently, Hematoxylin and Eosin (H&E) were used to stain the 5µm thick slices of stomach tissue. In 10 independent high-power fields (40 x), the damage to the stomach mucosa and submucosa was evaluated, as stated by Bancroft & Gamble [32]. The pathological markers employed for the assessment of the stomach injury were inflammatory cellular infiltrates (score: 0–3), bleeding (score: 0–4), and epithelial cell loss (score: 0–3). These three half scores add up to the overall pathologic score.

#### 2.4.6. Immuno-histochemical studies of caspase-3 and TLR-4.

The test for immunohistochemistry was used to determine the expression of caspase-3 within the stomach tissues. Firstly, the paraffin-embedded stomach tissue pieces were rehydrated and waxed using alcohol. After that, the slices were kept in a 3% hydrogen peroxide condition to inhibit the body’s natural peroxidase activity. After that, tissues were handled with rabbit polyclonal anti-TLR4, 1:50, Santa Cruz, CA, USA, and rabbit monoclonal anti-caspase-3 (EPR 18297) (ab 184787) (abcam). DAB (diaminobenzidine) was used to visualize the immunological response. Ten randomly chosen high-power fields (40X) were used to semi-quantitatively assess the immunological response according to the proportion of positively colored cells as stated by El-Gendy et al.2023 [6]. A scale of 0–3 was used to score the results; 1 represented positive staining in 30% of the cells or HPF, 2 represented positive coloring in 30% to 70% of the cells or HPF, and 3 represented positive coloring in more than 70% of the cells or HPF.

#### 2.4.7. Biochemical analysis (stomach homogenate preparation).

A polytron homogenizer operating at 40 °C was utilized to create a 10% homogenate in 0.05 M phosphate buffer (pH 7). The homogenate was centrifuged for 20 min at 10,000 rpm to extract the mitochondria, erythrocytes, nuclei, and broken cells. The cytoplasmic extract, or supernatant, was utilized to measure several biochemical parameters according to manufacturer’s instructions.

#### 2.4.8. Measuring signs of oxidative stress.

Oxidative stress markers were estimated in stomach homogenates utilizing Bio Diagnostic Company products for MDA enzymatic colorimetric analysis and GSH at wavelength 534 nm based on the procedure of Ohkawa, *et al*. [33] and 405 nm based on the procedure of Tietze [34], respectively.

#### 2.4.9. Enzyme linked immunosorbent assay (ELISA).

The protein content of the tissue was assessed using the Bradford technique [35]. Protein levels of NLRP3 were determined using a Genei protein estimation kit from Aviva Systems Biology (Catalogue No.: KD0290), Daxing Industrial Development Zone, Beijing, China. MyD88 levels were measured using a Fine Test kit (Catalogue No.: ER1598) from Fine Test Company, Eastlake High-tech Development District, Wuhan, Hubei, China. TNFα levels were quantified using an ELISA kit from Elabscience Biotechnology Co., Ltd, USA (Catalogue No.: 2.4.10). Each ELISA kit was measured using an ELISA plate reader (Stat Fax 2200, Awareness Technologies, Florida, USA) at an optical density (OD) range of 490–630 nm.

#### 2.4.10. Western blot assay of Nuclear Factor Kappa B one (NF-κβ), HMGB1.

Following the manufacturer’s instructions, every uniform tissue sample was processed with the Ready PrepTM protein extraction kit (total protein) from Bio-Rad Inc. (Catalogue #163–2086). Bradford Protein Assay Kit (SK3041) for quantitative protein analysis was supplied by Bio Basic Inc. (Markham, Ontario, L3R 8T4 Canada). Each sample containing 20 μg of protein was mixed with an equal volume of 2x Laemmli sample buffer, which consisted of 4% SDS, 10% 2-mercaptoethanol, 20% glycerol, 0.004% bromophenol blue, and 0.125 M Tris HCl. The mixtures were then heated at 95°C for 5 min to ensure complete protein denaturation before loading onto the polyacrylamide gel for electrophoresis.

The blot was then conducted on the BioRad Trans-Blot (Santa Cruz Biotechnology, Inc. California, USA) Turbo for 7 min at 25 V; to allow protein bands to transfer from gel to membrane in tris-buffered saline containing 3% bovine serum albumin (BSA) and Tween 20 (TBST) buffer, the membrane was occluded for one hour at normal temperature. The blocking buffer was composed of 20 mM Tris pH 7.5, 150 mM NaCl, 0.1% Tween 20, and 3% bovine serum albumin (BSA). As per the guidelines provided by the manufacturer, NF-κβ and HMGB1 primary antibodies were diluted for TBST (Santa Cruz Biotechnology, California, USA, www.scbt.com). Each primary antibody solution was left to incubate on the blotted target protein overnight at 4 °C. The blot was washed three times with TBST for 5 min. The HRP-conjugated secondary antibody solution was treated with the blotted target protein (Goat anti-rabbit IgG-HRP-1mg Goat mab - Novus Biologicals) for one hour at normal temperature. For five minutes, the blot was washed three times with TBST.

Per the manufacturer’s directions, the chemiluminescent substrate (ClarityTM Western ECL substrate, Bio-Rad cat#170–5060) was applied to the blot. The same quantities of the Clarity Western luminal/enhancer solution (solution A) and the peroxidase solution (solution B) were applied. The imager that recorded the chemiluminescent signals was based on a CCD camera. Using protein normalization on the Chemi Doc MP imager, image analysis software was used to evaluate the band intensity of the target proteins relative to the housekeeping protein, β-actin. [36].

### 2.5. Ultra-high-performance liquid chromatography coupled to electrospray ionization quadrupole time-of-flight mass spectrometry (UHPLC-ESI-qTOF-MS/MS) profiling of *P. maritimum*

The protocol that was previously described by Otify *et al.,* [37] was followed in the current study. A Waters ACQUITY I-Class UHPLC system with a Binary Solvent Module and FL Sample Manager was used to inject 2 µL of the tested sample partially, using a method of partial injection. The Waters ACQUITY UHPLC BEH C-18 column (2.1 mm i.d × 50 mm length, 1.7 µm particle size, Waters GmbH, Eschborn, Germany) was then used to perform the chromatographic separation at a flow rate of 300 µL/min at 55 °C. Water (A; BarnsteadTM GenPureTM, Thermo ScientificTM), CH_3_CN (B; ChromasolvTM, for LC-MS, Honeywell Riedel de HaënTM), and formic acid (0.1%additive for LC-MS, LiChropur®, Merck) comprised the eluting system. In order to apply the gradient elution, solvent B (3%, 1 min, isocratic) was added, rising to 95% B (within 7 min), then at 95% B (3 min), and finally, the column was re-equilibrated with 3% B (2.5 min). A hybrid qTOF mass spectrometer (Sciex TripleTOF 6600 LC-MS System, AB Sciex, Darmstadt, Germany) operating in negative ion mode was online infused with the column effluents. The ion spray voltage was operated at -4500 V, while the source temperature was maintained at 450 °C. Adjustments were made to the nebulizer, drying, and curtain gases at 85, 70, and 55 psig, respectively. MS specifications included: TOF-scan mode (accumulation time: 100 ms) was set at m/z ranging from 50 to 1500. 50 ms accumulation time at the collision potential (CE) of -40 V, collision energy spread (CES) of 10 V, and declustering potential (DP) of -35 V, the MS2 information-dependent acquisition (IDA) mode experiments (mass tolerance 25 ppm, m/z 50–1500, intensity > 100, exclude isotope window 4 Da) were completed. The collision activation dissociation (CAD) gas used was nitrogen.

### 2.6. Statistical analysis

The data were presented as mean ± Standard error (SE). One-way analysis of variance (ANOVA) was carried out using the Graph Pad Prism 8 software (Graph Pad, San Diego, CA, USA) and Tukey’s post-hoc test for group comparisons. the difference was considered significant at p <0.05.

## 3. Results

### 3.1. Acute toxicity of PM-EtOH extract

The rats treated with the plant extract showed no mortality or toxicity signs during this experiment. There were no modifications in body weight, aberrant physiological changes, or behavioral abnormalities at 2 g/kg dosages over 7 days. The rats showed no significant symptoms of toxicity at the orally administered doses.

### 3.2. Biological results

#### 3.2.1. Effect of PM-EtOH extract against EtOH-induced gastric ulcer.

**Fig 1:**
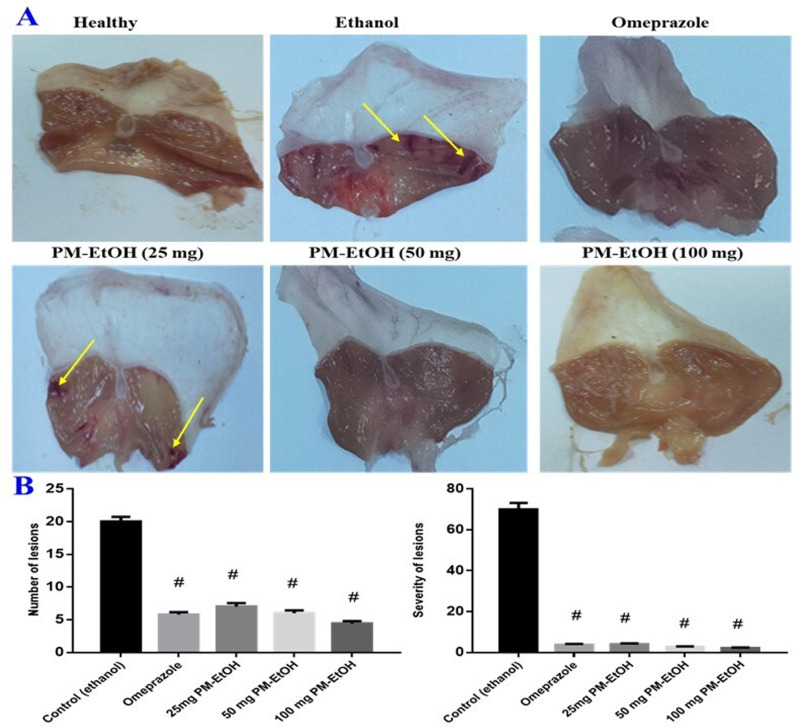
Macroscopic results of the protective impacts of *P. maritimum* extract (PM-EtOH) on ethanol induced-stomach mucosal damage in rats. (A) The normal group had no macroscopic lesions. Bands of hemorrhage were the result of significant lesions caused by ethanol to the control group’s stomach mucosa. Omeprazole therapy (20 mg/kg) or PM-EtOH (25, 50, 100 mg/kg) notably decreased the lesions caused by gastric mucosal bleeding respectively, and (B) Ethanol induced gastric lesions. Each bar symbolizes the mean of 6 rats. SE; # ****P**** < 0.05 vs. (control group), using one-way ANOVA then Tukey-Kramer multiple comparisons test.

The impact of PM-EtOH on the number of lesions in the stomach and severity induced by ethanol was determined. No macroscopic lesions were detected in the normal group. In ulcer-control group, there were severe gastrointestinal mucosal injuries, such as hyperemia as well as linear bleedings (**Fig 1A**), with a number of lesions 20 ± 0.71 and severity of lesions 70 ± 3.16. In contrast, PM-EtOH pretreatment at three dosage levels: 25, 50, and 100 mg significantly reduced the numbers of lesions at 7 ± 0.55, 6 ± 0.45, 4.4 ± 0.4 and severity as 4 ± 0.45, 2.6 ± 0.24, 2.2 ± 0.2 compared to ethanol-induced group, within the same circumstances, omeprazole therapy significantly decreased number and severity as 5.8 ± 0.3 and 3.8 ± 0.3 respectively, after comparison with the control group (**Fig 1B**).

#### 3.2.2. Effect of PM-EtOH extract on stomach morphological changes induced by EtOH.

**Fig 2:**
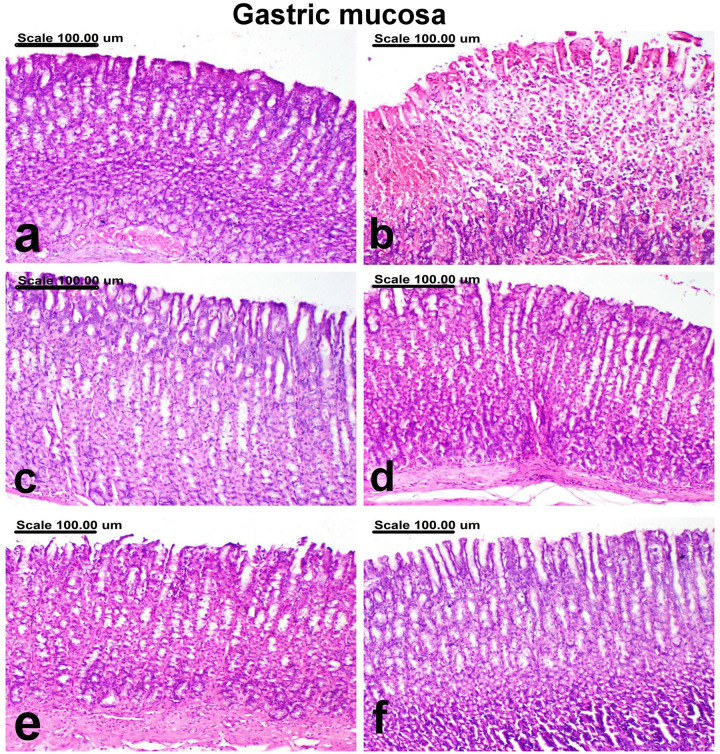
A photomicrograph of the gastric mucosa. (a) normal control group showing normal gastric mucosal epithelium, (b) ethanol group showing extensive necrosis and desquamation of mucosal epithelium associated with hemorrhage, (c) Omperazole group showing an attempt repair with proliferation of the gastric tubular glands, which appeared basophilic, (d) PM-EtOH (25 mg/kg) group showing normal gastric mucosal glands, (e) PM-EtOH (50 mg/kg) group showing necrosis of the superficial epithelial cells, (f) PM-EtOH (100 mg/kg) group showing regeneration of the gastric mucosal glands, which are lined with large basophilic vesicular nuclei. (Stain: H&E; Scale bar= 100µm).

**Fig 3:**
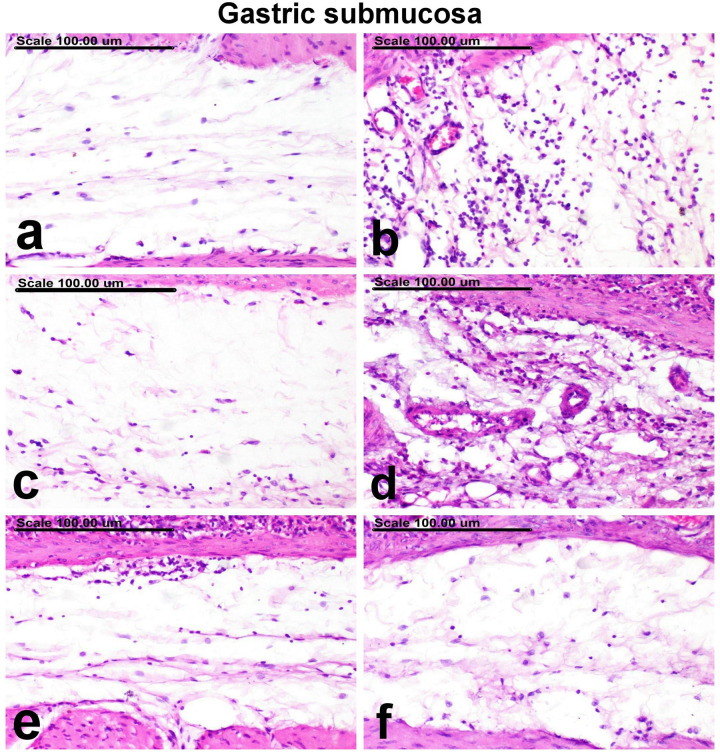
A photomicrograph of the gastric submucosa. (a) normal control group showing normal submucosa, (b) ethanol group showing intense infiltration of the submucosa with inflammatory cellular infiltrates, (c) Omperazole group showing no inflammatory cellular infiltrates in the submucosa, (d) PM-EtOH (25 mg/kg) group showing mild infiltration of the submucosa with inflammatory cells, (e) PM-EtOH (50 mg/kg) group showing minimal infiltration of the submucosa with inflammatory cells, (f) PM-EtOH (100 mg/kg) group showing normal submucosa. (Stain: H&E; Scale bar= 100µm).

**Table 1** reveals the total pathological score for each group’s stomach damage. The stomachs of the normal group showed normal sub-mucosa, normal gastric mucosal epithelium, and normal gastric glands (**Fig 2a** and **3a**). On the contrary, severe pathological alterations were demonstrated in the ulcer-control group, represented by extensive necrosis and desquamation of mucosal epithelium associated with hemorrhage (**Fig 2b**). The sub-mucosa of this group revealed sub-mucosal edema and intense inflammatory cellular infiltrates (**Fig 3b**). There was a notable improvement *via* a decline in the total pathologic score in the groups treated with omeprazole as well as other treatment groups. The stomachs of the omeprazole group revealed a clear repair, with proliferation of the gastric tubular glands, which appeared basophilic (**Fig 2c**). In this group, there were no signs of inflammatory cellular infiltration in the submucosa (**Fig 3c**). A pronounced improvement was recorded in groups treated with PM-EtOH, with a reduction in the severity of histopathological lesions which appeared as improvement in histopathological features. Normal gastric mucosal glands and mild infiltration of the sub-mucosa with inflammatory cells were demonstrated in most examined sections of 25 mg/kg of PM-EtOH group (**Figs 2d** and **3d**). Focal erosion with necrosis of the superficial epithelial cells and limited inflammatory cell infiltration of the submucosa were characteristic features of 50 mg/kg of PM-EtOH group (**Figs 2e** and **3e**). Individual apoptosis and regeneration of the gastric mucosal glands, which are lined with large basophilic vesicular nuclei, in addition to normal sub-mucosa, were detected in 100 mg/kg of PM-EtOH group (**Figs 2f** and **3f**).

**Table 1:** The total pathologic score for each group’s stomach damage.

Overall pathologic score (mean ± SE)	Groups
0.20^e^ ± 0.13	Normal control
6.70^a^ ± 0.21	Ulcer control group
1.10^d^ ± 0.31	Omeprazole
3.60^b^ ± 0.17	25 mg/kg PM-EtOH
2.40^c^ ± 0.42	50 mg/kg PM-EtOH
0.90^d,e^ ± 0.17	100 mg/kg PM-EtOH

SE: Standard error (±); Means followed by different letters indicating significance. ^*^Significant differences at *P < 0.05*

#### 3.2.3. Effect of PM-EtOH extract on stomach TLR4 and Caspase-3 immunohistochemical expression.

**Fig 4:**
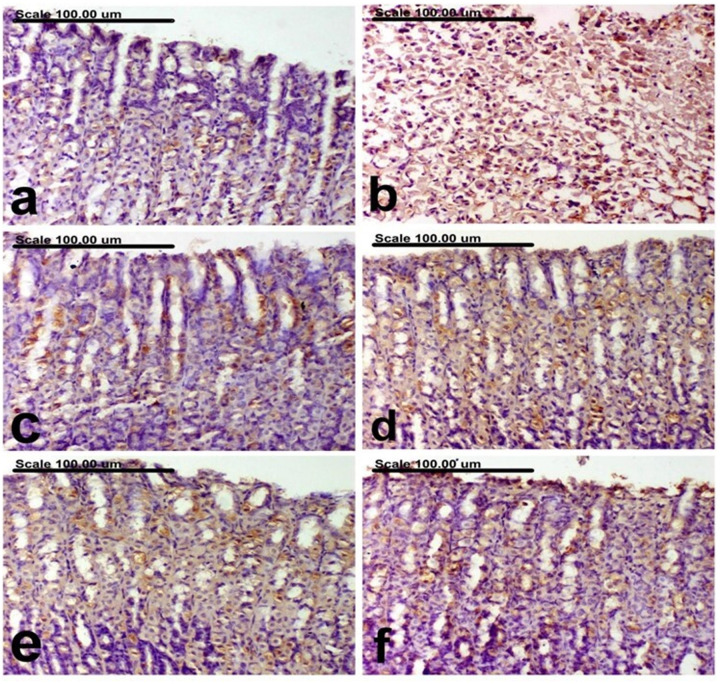
An immunohistochemically stained photomicrograph of the stomach mucosa using an anti-TLR4 antibody. (a) few TLR4-positively stained cells in the normal group; (b) the control ethanol group showed elevated TLR4 expression and a higher proportion of positive cells having deep brown coloring, (c) Omeprazole group showed reduction in the percentage of TLR4 positive cells with strong brown coloring, (d) PM-EtOH (25 mg/kg) group showed lowered percentage of TLR4 positive cells with weak cytoplasmic staining, (e) PM-EtOH (5o mg/kg) group showed decreased percentage of TLR4 positive cells, (f) PM-EtOH (100 mg/kg) group giving weak TLR4 expression, with a significant decrease in TLR4-positively stained cells(TLR4 immunohistochemical staining; Scale bar=100µm).

**Fig 5:**
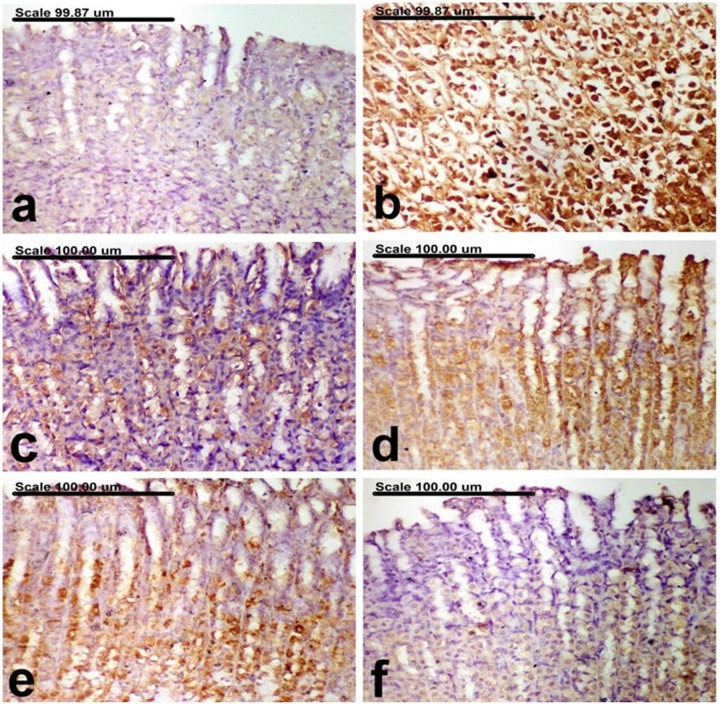
Photomicrograph of the gastric mucosa colored with an anti-caspase-3 antibody. The percentage of caspase-3 positive cells having strong brown coloring is reduced in the omeprazole group (c), while the control ethanol group (d) exhibited increased expression of caspase-3 and a higher percentage of positive cells with powerful brown cytoplasmic and/or nuclear coloring, (d) PM-EtOH (25 mg/kg) group showing decreased expression of caspase-3, along with the presence of moderately stained cells, (e) PM-EtOH (50 mg/kg) group showing decreased percentage of caspase-3 positive cells, (f) PM-EtOH (100 mg/kg) group showing weak caspase-3 expression, via a significant lowering in caspase-3-positively colored cells. (Caspase-3 immunohistochemical coloring; Scale bar= 100µm).

**Table 2** summarizes the expression of TLR4 and caspase-3 in gastric tissues across different treatment groups. In normal rats, very few cells stained positively for TLR4 and caspase-3 (**Figs 4a and 5a**). However, in the ulcer control group, there was a significant increase in the expression of both markers, with a high percentage of cells showing intense brown staining (**Figs 4b and 5b**). In contrast, the expression of TLR4 and caspase-3 was significantly reduced in reference drug omeprazole (**Figs 4c and 5c**) and PM-EtOH-treated groups, particularly at 100 mg/kg, as evidenced by a lower percentage of cells stained positively. For treated rats, a dose-dependent decrease in TLR4 and caspase-3 expression was observed, with 25 and 50 mg/kg groups showing weak cytoplasmic staining (**Figs 4d and 4e**). Similarly, in these groups, caspase-3 expression was also reduced, with cells displaying moderate staining (**Figs 5d and 5e**). The 100 mg/kg group demonstrated weak expression of TLR4 and caspase-3, with a significant reduction compared to the ethanol group (**Figs 4f and 5f**).

**Table 2:** TLR4 and Caspase-3 expressions in the stomach tissues of treated and untreated groups.

Caspase-3 expression (% of positive cells/HPF	TLR4 expression (% of positive cells/HPF)	Groups
0.90^d^ ± 0.17	0.40^e^ ± 0.16	Normal control
2.90^a^ ± 0.10	2.50^a^ ± 0.22	Ulcer control group
1.10^c,d^ ± 0.17	0.80^c,d^ ± 0.20	Omeprazole
1.70^b^ ± 0.21	1.30^b^ ± 0.15	25 mg/kg PM-EtOH
1.60^b,c^ ± 0.22	1.30^b^ ± 0.26	50 mg/kg PM-EtOH
1.00^d^ ± 0.21	0.70^c,d^ ± 0.21	100 mg/kg PM-EtOH

SE: Standard error (±); Means followed by different letters indicating significance. ^*^Significant differences at *P < 0.005.*

#### 3.2.4. Effect of PM-EtOH extract on MDA, GSH, MyD88, NLRP3 &TNF-α levels.

**Fig 6:**
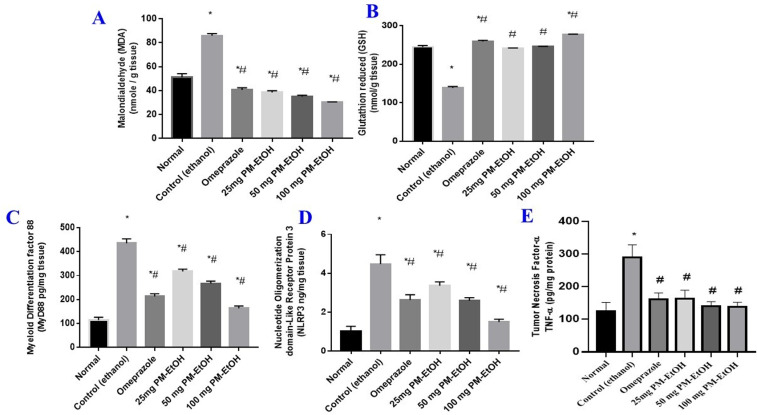
The effect of *P. maritimum* extract (PM-EtOH) on oxidative markers and inflammatory cytokines contents in rats with ethanol-induced stomach ulcers. (A) MDA, (B) GSH (C) MyD88, (D) NLRP3 and (E) TNFα. The mean of six rats is represented by each bar. SE; one-way ANOVA followed by the Tukey-Kramer multiple comparisons test; * ****P**** <.05 vs. (normal group) and # **P** <.05 vs. (control group).

Ethanol administration markedly increased MDA level (**Fig 6A**) simultaneously with a drop in GSH (**Fig 6B**) by 1.68 and 1.8 folds, respectively, after comparison with the normal group. In contrast, pre-medication in three doses of PM-EtOH (25, 50, and 100 mg/kg b.w.) markedly increased GSH levels concurrent with reduced MDA levels by 55.2%, 59.1%, 64.7% and 74.5%, 77.7%, 100.1% respectively, in comparison with the EtOH-induced group, in the identical circumstances, pre-treatment with omeprazole significantly reduced contents of MDA and increased that of GSH by 52.8% and 87.4%, respectively, after comparison with EtOH-induced group (**Fig 6A & B**).

Ethanol administration dramatically increased MyD88 (**Fig 6C**), NLRP3 (**Fig 6D**), and TNF-α levels (**Fig 6E**) by 3.8, 4.4, and 2.3 folds, respectively, in contrast to the normal group. In contrast, the three dosages of PM-EtOH administered 25, 50, and 100 mg/kg b.w. remarkably reduced the levels of MyD88, NLRP3, and TNFα by 27%, 38.5%, 62,4%; 24.8%, 42.2%, 66.5%, and 43.8%, 51.4%, 52.4% respectively, in contrast to the EtOH-induced group. In the same setting, omeprazole pretreatment dramatically reduced MyD88, NLRP3, and TNF-α by 51%, 41.3% and 44.4%, respectively, compared with EtOH-induced group.

#### 3.2.5. Effect of PM-EtOH extract on protein expression of HMGB1 and Nuclear Factor Kappa B (NF-κβ1).

**Fig 7:**
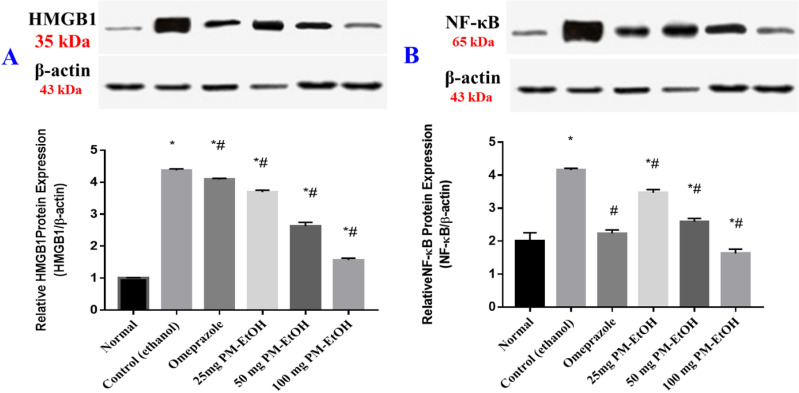
The effect of *P. maritimum* extract (PM-EtOH) on protein expression of (HMGB1) and (NF- κβ) in rats administered ethanol to develop stomach ulcers. The mean of six rats is represented by each bar. SE; one-way ANOVA followed by the Tukey-Kramer multiple comparisons test; * **P** <0.05 vs. (normal group) and # **P** <0.05 vs. (control group). The raw uncropped Western Blotting plates were presented as supporting data (Raw WB).

The administration of ethanol markedly elevated HMGB1 protein expression level (**Fig 7A**) and NF-кβ1 (**Fig 7B**) by 4.3 and 2.1 folds, respectively, in contrast to the normal group. In contrast, protein expression of HMGB1 and NF-κβ was considerably reduced by pre-treatment with PM-EtOH at the three dose levels of (25, 50, and 100 mg/kg b.w) by 15.5%, 39.8%, 64.1% and 16.5%, 37.7%, 60.8% respectively, with EtOH-induced group. In the identical setting, omeprazole pre-treatment dramatically reduced HMGB1 and NF-κβ protein expression by 6.5% and 46.1%, respectively, in comparison to the control group. (**Fig 7**).

### 3.3. UHPLC-ESI-qTOF-MS/MS metabolites profiling of *P. maritimum*

**Fig 8:**
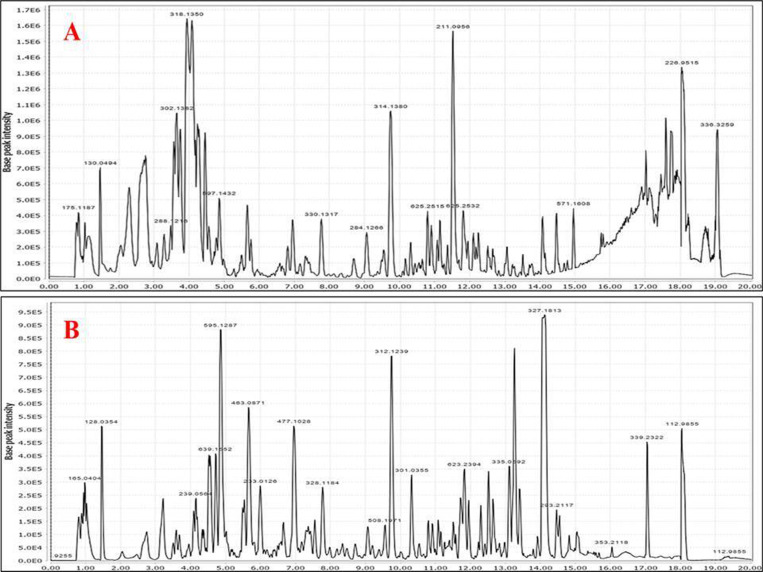
UHPLC-ESI-qTOF-MS/MS base peak chromatograms of *P. maritimum* ethanol extract (PM-EtOH). (**A**) positive ionization mode, and (**B**) negative ionization mode.

**Fig 9:**
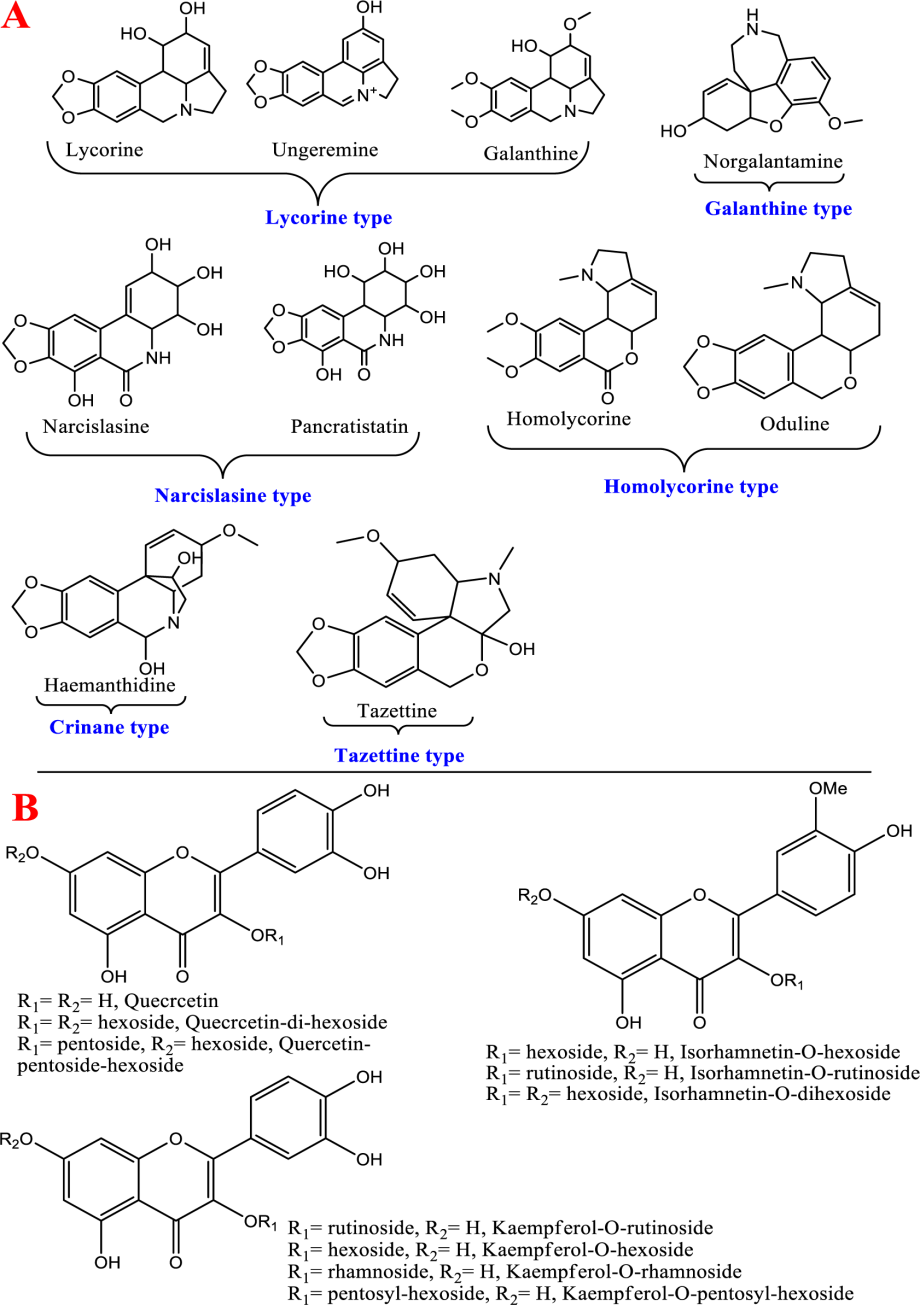
Representative chemical structures of major metabolites recognized in *P. maritimum* ethanol (PM-EtOH) extract *via* UHPLC-ESI-qTOF-MS/MS in negative and positive ion modes.

Eighty-four metabolites were annotated in PM-EtOH using both negative and positive ionization techniques (**Table 3 & Fig 8**). Metabolites were annotated *via* comparing their retention time, mass spectral data (molecular ion and daughter ions), and molecular formula with formerly published works and databases, like the GNPS library and the phytochemical dictionary of natural products. The identified phytochemicals and their chromatographic data are illustrated in **Table 3** and **Fig 8**. Metabolites were eluted in accordance with their polarity in positive and negative ion modes. Phytochemicals belonged to several different chemical groups, as amino acids, organic acids, phenolic acids, alkaloids, flavonoids, and fatty acids. Results revealed that both alkaloids and flavonoids were predominant in PM-EtOH. Positive ionization mode is considered a good representative for alkaloids, while most flavonoids were detected in negative ionization mode. Moreover, the chemical structures of major detected metabolites are displayed in **Fig 9**. Detailed interpretation of the putatively identified metabolites is described in the subsequent subsections.

**Table 3:** UHPLC-ESI-qTOF-MS/MS based characterization of secondary metabolites of the *P. maritimum* ethanol extract (PM-EtOH) in both positive and negative ionization modes.

No	R_t_	Compound name	Chemical class	[M-H]^-^/ [M^+^H]^+^	Molecular formula	Error (ppm)	MS^2^ fragments
1	0.84	Arginine	Amino acid	175.1185	C_6_H_15_N_4_O_2_^+^	-6.2	158
2	0.98	Xylonic acid	Sugar acid	165.0405	C_5_H_9_O_6_^-^	6.6	147, 129
3	0.98	Gluconic acid	Sugar acid	195.0510	C_6_H_11_O_7_^-^	0.226	129, 89, 75
4	0.99	Disaccharide	Sugar	377.0855	C_18_H_17_O_9_^-^	0.53	341, 215, 195
5	1.03	Maleic acid	Organic acid	115.0037	C_4_H_5_O_5_^-^	0.75	89
6	1.03	Hexosyl pyroglutamate	Amino acid	290.0883	C_11_H_16_NO_8_^-^	0.68	128
7	1.12	Galanthine	Lycorine-type alkaloid	318.1705	C_18_H_24_NO_4_^+^	1.25	300, 288, 271,268,258 193, 162
8	1.46	Citric acid	Organic acid	191.0196	C_6_H_7_O_7_^-^	0.5	173, 129
9	1.47	Coumaric acid	Hydroxy cinnamic acid	165.0544	C_9_ H_9_ O_3_^+^	-2.4	147, 123, 119
10	1.49	Chelidonic acid	Organic acid	185.0073	C_7_H_4_O_6_^+^	5.3	141, 97
11	1.52	Deoxy fructosyl leucine	Amino acid	294.1534	C_12_H_24_NO_7_^+^	-4.4	276, 258, 248, 230
12	2.36	Ungminorine-O-De-Me	Lycorine-type alkaloid	304.1170	C_16_H_18_NO_5_^+^	-5.4	286, 274, 256,245,244 227, 215,199, 177
13	2.72	Phenylalanine	Amino acid	166.0857	C_9_H_12_NO_2_^+^	3	120, 103
14	2.76	Norgalantamine	Galanthamine-type alkaloid	274.1424	C_16_H_20_NO_3_^+^	-6.9	231,213,198
15	3.2	Narcislasine	Narcislasine-type alkaloid	308.0750	C_17_H_27_NO_4_^+^	0.3	290, 272
16	3.2	Caffeic acid-*O*- glucuronide	Hydroxy cinnamic acid	355.0671	C_15_H_15_O_10_^-^	0.699	179, 85
17	3.22	Piscidic acid	Hydroxy cinnamic acid	255.0508	C_11_H_11_O_7_^-^	-0.9	193,165
18	3.48	Lycorine	Lycorine-type alkaloid	288.1217	C_16_H_18_NO_4_^+^	-3.8	270, 252, 227, 177
19	3.56	Indole-acrylic acid	Indole-type alkaloid	188.0701	C_11_H_10_NO_2_^+^	-4.6	170,146, 144, 118,115
20	3.59	Tryptophan	Amino acid	203.0824	C_11_H_11_N_2_O_2_^-^	2.9	188, 146
21	3.65	Oduline	Homolycorine-type alkaloid	302.1382	C_17_H_20_NO_4_^+^	-4.6	284,266,255,193
22	3.74	Incratine	Lycorine-type alkaloid	334.1631	C_18_H_24_NO_5_^+^	2.6	316, 302, 266, 284, 229, 191
23	3.8	Unknown	Unknown	372.1080	C_19_H_19_NO_7_^-^	3.14	298,211,126
24	3.82	Ferulyl saccharic acid	Hydroxy cinnamic acid	385.0777	C_16_H_17_O_11_^-^	1.8	209,191,147
25	3.94	Haemanthidine	Crinane-type alkaloid	318.1327	C_17_H_20_NO_5_^+^	5	286,268, 277,225, 211, 199
26	4.06	Tazettine	Tazettine- type alkaloid	332.1467	C_18_H_22_NO_5_^+^	-3.9	314, 300, 282, 264, 225, 199
27	4.16	Eucomic acid	Hydroxy cinnamic acid	239.0561	C_11_H_11_O_6-_	2.09	195,179,149, 107
28	4.19	Tetra hydro- *β*- carboline-carboxylic acid	*β*-carboline-type alkaloid	215.0827	C_12_H_11_O_2_N_2_^-^	-3.2	171,144
29	4.29	Homolycorine	Homolycorine-type alkaloid	316.1528	C_18_H_22_NO_4_^+^	-6.32	300, 286, 268, 227,207, 177
30	4.34	Pancratistatin	Narcislasine-type alkaloid	326.0857	C_14_H_16_NO_8_^+^	2.37	272, 244,224,222, 213, 208, 188
31	4.53	Roseoside	Norisoprenoid glycoside	431.1923	C_19_H_29_O_8_^-^	-1.02	385, 223, 205
32	4.54	Methoxy coumarin (herniarin)	Coumarin	177.0540	C_10_H_9_O_3_^+^	-4.5	162, 149, 145, 115
33	4.56	Quercetin-*O*- di hexoside	Flavonol-*O*-glycoside	625.1401	C_27_H_29_O_17_^-^	-1.5	461, 301
34	4.56	Ungeremine	Lycorine-type alkaloid	266.0799	C_16_H_12_NO_3_^+^	-6.7	236, 208, 180, 167
35	4.68	Orientin	Flavone-*C*-glycoside	447.0935	C_21_H_19_O_11_^−^	1.5	357, 327
36	4.72	Isorhamnetin-*O*-dihexoside	Flavonol-*O*-glycoside	641.1693	C_28_H_33_O_17_^+^	-2.7	479, 317
37	4.75	Narseronine	Homolycorine-type alkaloid	330.1319	C_18_H_20_NO_5_^+^	-5.14	272, 239, 227, 199
38	4.85	Quercetin-*O*-pentoside-hexoside	Flavonol-*O*-glycoside	595.1287	C_26_H_27_O_16_^-^	-1.87	301
39	5.06	Vitexin	Flavone-*C*-glycoside	433.1113	C_21_H_21_O_10_^+^	-3.8	415, 313, 271
40	5.2	Unknown	Unknown	277.1170	C_14_H_17_N_2_O_4_^+^	-4.23	231, 179,166,120
41	5.5	Kaempferol-*O*-rutinoside	Flavonol-*O*-glycoside	593.1505	C_27_H_29_O_15_^-^	-1.6	285
42	5.52	Kaempferol-*O*-pentosyl-hexosdie	Flavonol-*O*-glycoside	581.1486	C_26_H_29_O_15_^+^	3.8	449, 287
	5.54	Kaempferol-*O*-pentosyl-hexosdie	Flavonol-*O*-glycoside	579.1346	C_26_H_27_O_15_^-^	1.3	285
43	5.62	Isorhamnetin-*O*-rutinoside	Flavonol-*O*-glycoside	623.1608	C_28_H_31_O_16_^-^	2.24	315
	5.63	Isorhamnetin-*O*-rutinoside	Flavonol-*O*-glycoside	625.1752	C_28_H_33_O_16_^+^	0.6	479, 317
44	5.66	Quercetin-*O*-hexoside	Flavonol-*O*-glycoside	463.0876	C_21_H_19_O_12_^−^	0.4	301
45	5.96	Ferulic acid	Hydroxycinnamic acid	193.0503	C_10_H_9_O_4_^-^	1.07	178, 149, 134
46	6.11	Unknown	Unknown	306.0255	C_13_H_8_NO_8_^-^	1.46	260, 252,218,188
47	6.54	Unknown	Unknown	223.1317	C_13_H_18_O_3_^+^	6.28	205, 195,187, 181, 177, 121, 111
48	6.6	Kaempferol-*O*-hexoside	Flavonol-*O*-glycoside	447.0928	C_21_H_19_O_11_^−^	0.22	285
49	6.81	Loliolide	Terpene lactone	197.1166	C_11_H_17_O_3_^+^	-4.3	179, 161,107
50	6.95	Isorhamnetin-*O*-hexoside	Flavonol-*O*-glycoside	477.1030	C_22_H_21_O_12_^−^	-0.2	315,314
51	7.36	Oxo-ionol hexoside	Terpene glycoside	417.1967	C_19_H_32_O_7_^- +^ HCOOH	-0.8	371, 179
52	7.55	Azelaic acid	Fatty acid	187.0973	C_9_H_15_O_4_^-^	-1.87	143, 125
53	7.78	Ferulyl dopamine	Hydroxy cinnamic acyl amide	328.1184	C_18_H_18_NO_5_^-^	2.38	175
54	7.86	Unknown	Unknown	433.2312	C_19_H_29_N_8_O_4_^+^	0.33	416, 360, 300, 253, 207,179
55	8.34	Kaempferol-*O*-rhamnoside	Flavonol-*O*-glycoside	431.1339	C_21_H_19_O_10_^-^	-2.3	285
56	9.06	p-Coumaryl tyramine	Hydroxy cinnamic acyl amide	284.1263	C_17_H_18_NO_3_^+^	6.3	147
57	9.75	Ferulyl tyramine	Hydroxy cinnamic acyl amide	314.1371	C_18_H_20_NO_4_^+^	-3.6	177
58	10.32	Quercetin	Flavonol	301.0400	C_15_H_9_O_7_^-^	-0.34	273, 178, 151
59	10.53	Unknown	Unknown	211.1685	C_13_H_23_O_2_^+^	2.5	193, 175, 165, 135, 109
60	11.08	Unknown	Unknown	492.119	C_28_H_29_NO_7_^+^	6.3	462, 355, 337, 325
61	11.52	Trimethoxy acetophenone	Acetophenone	211.0694	C_11_H_15_O_4_^+^	-4.9	193, 180, 120, 103
62	11.7	Tri hydroxy octa-decanoic acid	Fatty acid	329.2300	C_18_H_33_O_5_^-^	-0.6	309, 293, 279, 211
63	11.71	Oxo-octadecadienoic acid	Fatty acid	295.2256	C_18_H_31_O_3_^+^	6.29	277, 259, 241, 189, 179
64	11.82	Grossamide	Benzofuran carboxamdie	623.2395	C_36_H_35_N_2_O_8_^-^	2.7	486, 460, 331, 291
65	11.95	Unknown	Unknown	195.0700	C_10_H_11_O_2_^-^	-0.3	180, 153, 120
66	12.18	Phytosphingosine	Sphingolipid	318.2989	C_18_H_40_NO_3_^+^	0	306, 285
67	12.21	Sakuranetin	Flavanone	285.0768	C_16_H_13_O_5_^-^	1.05	179, 165
68	12.24	Unknown	Unknown	181.1226	C_11_H_17_O_2_^+^	4.08	163, 135, 107
69	12.3	Unknown	Unknown	213.1100	C_11_H_17_O_4_^-^	-1.17	169, 96
70	12.52	Farrerol	Flavanone	299.0928	C_17_H_15_O_5_^-^	0.47	282, 205,179, 119
71	13.05	Trihydroxyacetophenone-Di-methyl ether	Acetophenone	197.0804	C_10_H_13_O_4_^+^	-0.8	179, 155
72	14.08	Hydroxyoctadecadienoic acid	Fatty acid	297.2061	C_18_H_33_O_3_^+^	0.9	279
73	14.15	Spiculisporic acid	Fatty acid	329.1954	C_17_H_29_O_6_^+^	2.4	265, 237, 209
74	14.46	Linolenic acid derivative	Fatty acid derivative	559.3113	C_28_H_47_O_11_^−^	1	277
75	14.5	Hyrdoxy linoleic acid	Fatty acid	293.2117	C_18_H_29_O_3_^-^	-0.46	277, 195
76	16.27	Unknown diterpene	Diterpene	441.3206	C_25_H_44_O_6_^+^	-0.8	421,315, 291, 263, 209
77	16.32	Linaloyl ethanolamide	Fatty acyl amide	324.2894	C_20_H_38_NO_2_^+^	1.2	306
78	16.94	Octadecatetraenoic acid	Fatty acid	277.2159	C_18_H_29_O_2_^+^	-0.78	261,243, 237, 223, 209, 173
79	16.95	Palmitoyl ethanolamide	Fatty acyl amide	300.2893	C_18_H_38_NO_2_^+^	-1.6	284, 283
80	17.16	Oleoyl ethanolamine	Fatty acyl amide	326.3036	C_30_H_40_NO_2_^+^	5.8	309
81	17.51	Unknown	Unknown	609.2711	C_34_H_41_O_10_^+^	1.23	592, 550, 416
82	17.59	Linolenyl alcohol	Fatty alcohol	265.2526	C_18_H_33_O^+^	6	247, 205, 191
83	17.83	Heptadecanamide	Fatty acyl amide	270.2789	C_17_H_36_NO^+^	-3	
84	17.97	Unknown	Unknown	269.2914	C_16_H_38_NO^+^	-8.9	279, 266, 265,238

R_t_*:* retention time

#### 3.3.1. Amino acids.

Five amino acids and their derivatives were eluted early at Rt. 0.84–3.59 min as illustrated in **Table 3 & Fig 8**. From which three amino acids and two derivatives were annotated in peaks **1**, **6**, **11**, **13**, and **20**. Arginine, phenylalanine (S1 Fig) and tryptophan were detected in peaks **1**, **13** and **20**, respectively [38]. In general, the major fragments of amino acids result from deamination (-NH_2_, 17 amu), decarboxylation (-CO_2_, 44 am), demethylation (-CH_2_, 14 amu) and dehydration (-H_2_O, 18 amu) of the molecular ion (S1 Fig) [39]. Peak **6** (*m/z* 290.0883, C_11_H_16_NO_8_^-^), showed a fragment ion at *m/z* 128 due to the loss of hexose moiety from the molecular ion yielding free pyroglutamic acid, and was assigned as hexosyl pyroglutamate [39]. One sugar acylated amino acid was shown in peak **11** *(m/z* 294.1534, C_12_H_24_NO_7_^+^) with MS/MS ions at *m/z* 276 [M+H-H_2_O] and 258[M+H-2H_2_O], annotated as deoxy fructosyl leucine (S2 Fig) [38]. Both hexosyl pyroglutamate and deoxy fructosyl leucine have been recognized firstly in *P. maritimum*.

#### 3.3.2. Organic acids.

Five organic acids were identified in PM-EtOH mainly in the negative ionization mode. Commonly, organic acids could be detected *via* the formation of some characteristic ions resulting from the decarboxylation and dehydration of the parent ion [40]. Notable identification included xylonic acid (peak 2, m/z 165.0405) [40], gluconic acid (peak 3, m/z 195.051)[38], malic acid (peak 5) and citric acid (peak 8) [41]. Malic acid and citric acid are common organic acids that contribute to organoleptic and antioxidant effects [42]. The fragmentation pattern of peak **10** (*m/z* 185.0073, C_7_H_4_O_6_^+^) was similar to that of chelidonic acid [43]. It showed daughter ions at *m/z* 141 [M+H-CO_2_] ^-^ and *m/z* 97[M+H-2CO_2_] ^-^ previously reported in the *Pancratium* genus.

#### 3.3.3. Phenolic acids and their derivatives.

Nine phenolic acids along with their derivatives were detected in both positive and negative ionization modes mainly hydroxy cinnamic acids either in their free form, or attached to sugar moiety, or aliphatic monoamines as illustrated in **Table 3**. In general, phenolic acids are detected *via* the loss of carboxylate group (-44 amu) to provide [M-H-COO] ^–^ ion [44], besides the loss of methyl radical (-15 amu) for methoxylated phenolic acids to yield [M-H-CH_3_]^-^ ion as in ferulic (peak 45, m/z 193.0503) [45]. Peaks **9** and **17** were annotated as p-coumaric acid [38] and Piscidic acid (S3 Fig) [46], respectively. Herein, piscidic was reported for the first time in PM-EtOH. Peak **16** (*m/z* 355.0671) showed a fragment ion at *m/z* 179 which assigned to a caffeoyl moiety [39] confirming the attachment of the glucuronide moiety (176 amu) to caffeic acid, and identified as caffeic acid-*O*-glucuronide [47]. Peak **24** was tentatively annotated as feruloyl saccharic acid (*m/z* 385.0777) [48], while peak **27** was identified as eucomic acid (*m/z* 239.0561) [49]. Caffeic acid-*O*-glucuronide, feruloyl saccharic acid, and eucomic acid are documented for the first time in genus *Pancratium.* Furthermore, UPLC analysis of PM-EtOH revealed the detection of 3 hydroxy cinnamoyl amides (HCAAs) belonging to phenolic acids attached to aliphatic monoamines or aryl polyamines. Feruloyl and *p*-coumaryl substituted amines are the commonly identified HCAAs in PM-EtOH detected at retention time from 7.78 to 9.75 min [50]. Peak **55** displayed M-H ^-^ at *m/z* 328.1184 with MS^2^ ion at *m/z* 175 for the feruloyl moiety resulting from the amide bond cleavage and the loss of dopamine (-153 amu) [50], and assigned as feruloyl dopamine. Following the same fragmentation pattern, peaks **56** (*m/z* 284.1263, C_17_H_18_NO_3_^+^) and **57 (***m/z* 314.1371, C_18_H_20_NO_4_^+^) were annotated as *p*-coumaryl tyramine and ferulyl tyramine (S4 Fig).

#### 3.3.4. Alkaloids.

Family Amaryllidaceae is known to accumulate alkaloids to which various pharmacological activities are attributed [51]. Fifteen alkaloids and their derivatives have been tentatively recognized in PM-EtOH as shown in **Table 3**. Some alkaloids’ chemical structures are displayed in **Fig 9**. These alkaloids showed different skeletons with diverse fragmentation patterns limiting level of confidence in assignment. There were 6 main groups of alkaloids identified based on their structures which are lycorine, homolycorine, galanthamine, narislasine, tazettine, and crinine types, in addition to few alkaloid derivatives. The MS/MS fragmentation data revealed the detection of 5 lycorine type alkaloids in peaks **7**, **12**, **18**, **22**, and **34**. MS/MS fragmentation data of this type showed key fragments resulting from the RDA cleavages of ring B & C (S5 Fig). Other fragment ions derived from the successive loss of substituents from ring B [52]. On this context, peak **18** (*m/z* 288.1217), accompanied with key fragment ion at *m/z* 227 due to RDA cleavage of ring B with the loss of (-CHOH=CH_2_OH) and two fragments at *m/z* 270 [M + H-H_2_O]^+^ and 252 [M + H-2H_2_O]^+^ (**Table 3 &** S5 Fig), was assigned as lycorine [53]. Following the same fragmentation pattern of lycorine, peaks **7**, **22** and **34** were annotated as galanthine, incratine, and ungeremine, respectively [53]. Peak **12** (*m/z* 304.117, C_16_H_18_NO_5_^+^) showed successive loss of two hydroxyl groups of ring B at *m/z* 286 and 268, with fragment ion at *m/z* 243 (RDA cleavage) that matched with ungminorine alkaloid [54] considering a mass difference of 14 amu (CH_3_ radical), so it was annotated as ungminorine-*O*-De-Me. It’s the first time for the detection of ungminorine-*O*-De-Me in PM-EtOH. Three homolycorine alkaloids appeared in peaks **21** (*m/z* 302.1382), **29 (***m/z* 316.1528), and **37** (*m/z* 330.1319), and were identified as oduline, homolycorine and narseronine, respectively [53]. Norgalanthamine alkaloid (S6 Fig) was observed in peak **14** (*m/z* 274.1424) [53]. Peak **25** (*m/z* 318.1327, C_17_H_20_NO_5_^+^) and **26** (*m/z* 332.1467, C_18_H_22_NO_5_^+^) were annotated as haemanthidine (S7 Fig) and tazettine [53]. Moreover, two narcislasine alkaloids were detected in peaks **15** & **30** assigned as narcislasine [55] and pancratistatin [56]. Furthermore, peak **19** (*m/z* 188.0701, C_11_H_10_NO_2_^+^) showed MS^2^ ion at *m/z* 170, 142, and 115 was assigned as indole-acrylic acid [57]. Peak **28** (*m/z* 215.0827, C_12_H_11_O_2_N_2_^-^) with daughter ions at *m/z* 171 (-CO_2_) & 144 (RDA fragmentation) was annotated as a tetrahydro-*β*-carboline-carboxylic acid [58]. Both indole-acrylic acid and tetra hydro-*β*-carboline-carboxylic acid were first time detected in *P. maritimum*. Amaryllidaceous alkaloids, galanthamine, lycorine, narcislasine, and crinamine were reported to inhibit cyclooxygenase, NFκB activation, nitric oxide, and p-38 MAP kinase which are the key mediators of the inflammation process [59].

#### 3.3.5. Flavonoids.

Polyphenolics especially flavonoids amounted for the second major secondary metabolite class in many amaryllidaceous plants belonging to flavonols, flavones, flavanones, isoflavones, chalcones, and flavans types. Fifteen flavonoids were tentatively detected in ionization modes that are both positive and negative belonging to *O*-glucosyl flavonols as the most abundant. Regarding the nature of the attached sugar, it could be concluded through the respective loss of 162 amu, 132 amu, and 146 amu corresponding to hexose, pentose, and deoxyhexose [39]. Quercetin-*O*-glycosides were detected in peaks **33**, **38**, and **44** and assigned as quercetin-*O*-di-hexoside, quercetin-*O*-pentosyl-hexoside, and quercetin-*O*-hexoside, respectively [20]. Concerning isorhamnetin-*O*-glycosides, peaks **36**, **43**, and **50** were assigned as isorhamnetin-*O*-di-hexoside, isorhamnetin-*O*-rutinoside, and isorhamnetin-*O*-hexoside (S8 Fig), respectively [20] based on aglycone fragment ion of isorhamnetin at *m/z* 315. Likewise, kaempferol-*O*-glycosides were detected in peaks **41**, **42**, **48**, & **55** assigned as kaempferol-*O*-rutinoside (S9 Fig), kaempferol-*O*-pentosyl-hexoside, kaempferol-*O*-hexoside & kaempfeol-*O*-rhamnoside, respectively [20]. Flavonoid-*C*-glycoside exhibited a different fragmentation pathway resulting from cross ring cleavage of the sugar part with the loss of (−120 amu) and (-90 amu) for *C*-hexosides and (-60 amu) for *C*-pentosides [60] and detected in peaks **35** & **39**. The identified flavonoid-*C*-glycosides were flavone derivatives of luteolin and apigenin. For example, peak **35** (*m/z* 447.0935, C_21_H_19_O_11_^−^) with subsequent MS/MS ions at *m/z* 357 [M-H-90]^-^, and 327 [M -H-120]^-^ and annotated as orientin (luteolin-8-*C*-glucoside) [60], and likewise for vitexin in peak **39** [60]. This is the first report of orientin and vitexin in genus *Pancratium.* Compared to the abundance of flavonol subclass, flavanones appeared only in peaks **67** & **70**. Peak **67** (*m/z* 285.0768, C_16_H_13_O_5_^-^) with fragmentation behaviour as reported in sakuranetin [61] and reported first time in genus *Pancratium*. Peak **70** *(m/z* 299.0928) was identified as farrerol (S10 Fig) [62]. In general, flavonoids are known to exert anti-inflammatory and anti-oxidant activities. But still, quercetin was known to exhibit anti-inflammatory and gastroprotective activities *in vivo* [63].

#### 3.3.6. Fatty acids/amides and shingolipids.

A total of 8 fatty acids and their derivatives were detected at Rt >10.00 min of the chromatogram. Peaks **52**, **62**, **63**, **72**, **73**, **74**, **75**, and **78** belonged to fatty acids, while fatty acyl amides appeared in peaks **77**, **79**, **80**, and **83**. Hydroxylated fatty acids were determined by the absence of H_2_O (18 amu) and carboxylate (44 amu) moieties from the main skeleton [39]. Saturated fatty acids included azelaic acid (peak 52), tri hydroxy octadecanoic acid (peak 62) and spiculisporic acid (peak 73) [39]. Unsaturated fatty acids were annotated as oxo-octadecadienoic acid (peak 63), hydroxyoctadecadienoic acid (peak 72), hyrdoxy linoleic acid (peak 75) and octadecatetraenoic acid (peak 78) [38,64]. Fatty acyl amide is a characteristic fatty acid subclass that was detected for the first time in *P. maritimum*, and suggestive that nitrogen is incorporated in lypophilic classes’ asides from the more polar alkaloids. The chemical structure of fatty acyl amide is indicated by the fatty acid moiety’s amide bond connection to ethanolamine [38] as evident from the loss of ammonia (-17amu). Accordingly, peaks **77**, **79**, **80,** and **83** were assigned as linaloyl ethanolamide, palmitoyl ethanolamide, oleoyl ethanolamine and heptadecanamide [38]. One sphingolipid was detected the first time in the genus *Pancratium* appeared in peak **66** and was tentatively identified as phytosphingosine [65].

#### 3.3.7. Other compounds.

Other metabolite classes were also detected in PM-EtOH*.* Peak **31** showed M-H^-^ at *m/z* 431.1923 with fragment ion at *m/z* 385 due to loss of HCOOH and other MS^2^ ions similar to the fragmentation roseoside [66]. One terpene lactone appeared in peak **49** with [M+H]^+^ at *m/z* 197.1166 was assigned as loliolide [67]. Peak **51** was annotated as oxo-ionol hexoside [68]. Peak **64** with [M-H]^-^ at *m/z* 623.2395 was annotated as grossamide [69]. Roseoside, oxo-ionol hexoside, loliolide, and grossamide are reported first time in genus *Pancratium*. Two acetophenones appeared in peaks **61** & **71** assigned as tri-methoxy acetophenone (S11 Fig) and trihydroxyacetophenone-di-methyl ether [70].

## 4. Discussion

Stomach ulcer is a common digestive disorder that, if left untreated, can progress to more serious conditions, including stomach cancer [71]. In short, stomach ulcers result in a compromised state of health for the body alternative remedies are warranted for development as current medications do not offer complete safe cure [72]. Recently, efficient, safe, and widely available herbal treatments for stomach ulcers have emerged [73].

As ethanol-induced gastric ulcers resemble human ulcerative conditions in many ways, they might be useful to confirm the investigated agent’s anti-ulcer properties and the most feasible pathways through the three aspects of apoptosis, inflammation, and oxidative stress. Other models, such as indomethacin or pyloric ligation, were not chosen for this purpose [74].

Rats given ethanol in the current study showed more widespread mucosal lesions, edema, and apoptotic cells. The lesions have been verified histologically by H&E stain, which revealed thorough obliteration of all gastrointestinal layers, along with inflammation, constriction of blood vessels, and infiltration of mononuclear cells in the sub-mucosa. According to [75], ethanol is a necrotizing agent that may easily penetrate the gastric mucosa and cause damage to the vascular system, leading to gastric ulcers. It also has a direct toxicity on the epithelium as a result of neutrophil infiltration in the tissue surrounding the stomach ulcer. In contrast, compared with the standard medication, omeprazole, a seven-day *P. marfimum* treatment ameliorated ethanol-induced stomach damage and promoted gastric recovery, along with decreased histopathological changes, apoptotic marker caspase-3, and an influx of leucocytes, indicating its anti-ulcer actions.

Our results showed that ethanol caused stomach mucosal damage by upsetting the oxidant/antioxidant equilibrium, which is consistent with other studies [76]. Considering that ethanol is connected to purine disintegration, it results in an excess of reactive oxygen species (ROS) being produced and triggering oxidative damage such as cell death, lipid peroxidation, and epithelial damage [77]. The elevated level of the lipid peroxidation marker malondialdehyde (MDA) and the concurrent reduction in GSH indicated a failure to neutralize free radicals derived from oxygen and lipids in a process to create lipid peroxides. These peroxides are recognized as a reason for the degradation of ion transport and membrane integrity, loss of membrane fluidity, and ultimately a diminution of cellular activity that comes in agreement with [78]. Conversely, treatment using PM-EtOH preserved GSH content and a decreased level of MDA in the stomach, suggesting its antioxidant action.

An essential component of the immune system, the NLRP3 inflammasome promotes the creation and activation of many inflammatory factors [79]. Both infections and endogenous stress can promote inflammation cascade. Following an inflammasome activation, autocatalytic initiation of caspase-1 facilitates the cleavage of pro-inflammatory cytokines to release mature inflammatory cytokines, for instance, TNF-α, which consequently initiates the inflammatory response [80].

Alzokaky *et al.,* [14] revealed that HMGB1 is crucial to the healing of stomach ulcers. Chen et al., [81] reported that HMGB1, a molecular pattern molecule linked to cause damage (DAMP) that mediates inflammation and immune responses through TLR-4, is a pro-inflammatory mediator that is typically found in the nucleus and attaches itself to chromatin. However, under conditions of elevated reactive oxygen species (ROS), it actively and passively shuttles from the cytoplasm into the nucleus and finally into the extracellular area, where it exhibits pro-inflammatory activity. This increases the amount of TLR4 and MyD88 and follows the production of NF-к*β* to enhance the TLR4/MyD88/NF-κβ signaling pathway, producing TNF-α. [82]. TNF-α is known to impede stomach microcirculation surrounding the ulcerated mucosa, which diminishes the recuperation process, and in order to stimulate immunological cells through further NF-κβ stimulation [74].

These results are consistent with the mechanism by which ethanol in this study caused inflammation. Specifically, excessive generation of reactive oxygen species (ROS) activated the NLRP3 inflammasome, which in turn accelerated the lysine acetylation of HMGB1, promoted its translocation and release from nucleus to cytoplasm, triggering TLR4 and MyD88 content. This process was then followed by activating signaling pathways involving NF-κβ expression, which ultimately led to the production of TNF-α, resulting in inflammatory damage to the stomach [12,83].

According to studies, anti-inflammatory action plays a major role in preventing peptic ulcers. [84]. Manivannan and his co-workers [85] reported that HMGB1 might be a useful biomarker for brain damage, and inhibiting neuroinflammation could be a treatment strategy for several diseases. Consequently, down-regulation of HMGB1 expression may play a part in hastening the healing of stomach ulcers. Meng and his teamwork [86] revealed that inhibition of NF-к*β* and TNF-α production as pro-inflammatory cytokines, improves stomach ulcers caused by ethanol. Treatment with PM-EtOH resulted in significant down regulation of NLRP3 inflammasome and MGB1/TLR4/MYD88/NF-κβ signaling pathway. Comparable outcomes were noted in omeprazole treated rats. The aforementioned results indicated a novel anti-inflammatory agent for gastric ulcers.

Recently, significant progress in chemical and pharmacological investigations has expanded our understanding of novel therapeutically effective substances derived from natural sources. The chemical, toxicological, and medicinal characteristics of natural active component classes, such as flavonoids, tannins, alkaloids, terpenoids, and fatty acids, have drawn the attention of researchers [87]. Herein, we have reported the presence of several phytochemicals via UHPLC-ESI-qTOF-MS/MS belonging to alkaloids and flavonoids as major classes. The effect of 13 alkaloidal subclasses namely: imidazole, indole, isoquinoline, non-nitrogen heterocycle alkaloid, phenylalkylamide, piperidine, pyrazine, pyridine, pyrrolidine, pyrrolizidine, quinolizidine, steroid and tropane alkaloids, in preventing and releasing gastric ulcer was reported using both human and animal models [87]. In models of ulcers generated by both acetylsalicylic acid (ASA) and stress, intravenous injection of rutaecarpine alkaloid at doses of 100 or 300 mg/kg greatly lowered the ulcer’s severity grade and pH level in contrast to the placebo group [88]. 2-Phenylquinoline alkaloid hindered lesions caused by HCl/ethanol-induced ulcer model in rats, when administered at dose of 50 mg/kg [88]. As well, methoxycanthin-6-one alkaloid exhibited concentration-dependent preventive activity on gastric mucosa regarding indomethacin-induced gastric lesions in rats [88]. Alkaloids identified from *Mahonia bealei* possessed significant antiulcer activity mostly by impeding H+/K+-ATPase effect along with secretion of gastrin and gastric acid in pyloric ligation-induced rats [89]. Coptisine alkaloid exhibited anti-ulcer potential *via* inhibition of p38 MAPK and the activation of Nrf2 signaling pathway [90].

Conversely, flavonoids have been reported in several studies to possess both curative and preventive activity on gastric and intestinal epithelium. These activities may be attributed to preservation of the intestinal barrier, the uptake of lipids and carbohydrates, the alteration of enzyme activities, the controlling of stomach secretions, the immune system, and interactions with pathogenic microorganisms. The prospected mechanisms for the antiulcer activity of flavonoids comprise suppression of acid production, raising gastric mucus and bicarbonate secretion, as well as reducing the level and activity of pepsin. Moreover, flavonoids strengthen the antibacterial, anti-inflammatory, antioxidative, and cytoprotective defenses of the mucosa against peptic ulcers [91]. Herein, three main flavonoids and their glycosides were accompanied by mostly detected in plant extract: namely, quercetin, kaempferol, and isorhamnetin. Quercetin prevented oxidative stress and inflammation caused by indomethacin in gastric mucosa and Caco-2 cells by increasing the nuclear translocation of nuclear factor related to erythroid 2 (Nrf2) and the activity of glutathione peroxidase (GPx) and superoxide dismutase (SOD). Additionally, quercetin ameliorated ICAM-1 and P-selectin production in addition to nuclear factor kappa B (NF-κβ) activation brought on by indomethacin [92]. Kaempferol, another major flavonoid in PM-EtOH shields mice’s stomach sores evoked by ethanol by preventing neutrophil buildup, diminishing myeloperoxidase (MPO) activity, and lowering levels of pro-inflammatory cytokines like TNF-α, IL-1β, and interleukin-6 (IL-6), while also increasing gastric mucus and NO. At a minimum inhibitory concentration (MIC) of 0.05 mMol/L, kaempferol also inhibited *H. pylori* growth *in vitro*. AGS gastric cancer cells have diminished levels of TNF-α, IL-1β, and interleukin-8 (IL-8) as a result of this activity, which also includes the production of the genes for cytotoxin-associated gene A (CagA) and vacuolating cytotoxin A (Vac A) [92]. Using rats as the bioassay model, the butanol fraction of *Sambucus ebulus* L. leaves showed strong antiulcerogenic action against the stress ulcer model caused by immobilization and water immersion. Isorhamnetin glucoside and quercetin glucoside detected as major flavonoids in PM-EtOH were reported to inhibit the inflammatory pathway, in addition to their antioxidant effect [93] to mediate for the improved action in PM-EtOH treated animal group.

## 5. Conclusion

This study serves as the initial report of the effect of *Pancratium maritimum* L. towards ethanol-induced gastric lesions in rats *via* multiple action mechanisms. This healing effect may be explained by blocking the initiation of the HMGB1/TLR4/MYD88/NF-κβ signaling pathway, the NLRP3 inflammasome, and the suppression of apoptosis. This activity was attributed to several classes of phytochemicals in the extract identified via UHPLC-ESI-qTOF-MS/MS including alkaloids and flavonoids. This study suggests that PM-EtOH may be a novel, effective option in the management of ulcers. To thoroughly establish the dose-response relationship and identify the appropriate dosage required to achieve therapeutic effects, it is essential to conduct a comprehensive analysis. This includes determining the optimal dose through preclinical studies and validating the findings in clinical investigation to ensure the results are robust and conclusive. Furthermore, these findings should be compared against other ulcer models to evaluate the consistency and efficacy of the treatment across different experimental conditions. Such a multi-faceted approach ensures that the selected dosage is not only effective but also safe and reliable for clinical use. Individual compounds testing and standardization are essential actions in future research involving *P. maritimum* to be included in nutraceuticals for ulcer treatment.

## Supporting information

S1 Data Raw WB The raw uncropped Western Blotting plates. (DOCX)

S1 Table Gastric ulcer scoring system based on the severity of the ulcer. (DOCX)

S1 Fig MS/MS fragmentation pattern of phenylalanine. (DOCX)

S2 Fig MS/MS fragmentation pattern of deoxy fructosyl leucine. (DOCX)

S3 Fig MS/MS fragmentation pattern of piscidic acid. (DOCX)

S4 Fig MS/MS fragmentation pattern of ferulyl tyramine. (DOCX)

S5 Fig MS/MS fragmentation pattern of lycorine. (DOCX)

S6 Fig MS/MS fragmentation pattern of norgalanthamine. (DOCX)

S7 Fig MS/MS fragmentation pattern of haemnathidine. (DOCX)

S8 Fig MS/MS fragmentation pattern ofisorhamnetin-*O*-hexoside. (DOCX)

S9 Fig MS/MS fragmentation pattern of kaempferol-*O*-rutinoside. (DOCX)

S10 Fig MS/MS fragmentation pattern of farrerol. (DOCX)

S11 Fig MS/MS fragmentation pattern of trimethoxy acetophenone. (DOCX)
