# Supplementary material for: In vivo anti-ulceration effect of Pancratium maritimum extract against ethanol-induced rats via NLRP3 inflammasome and HMGB1/TLR4/MYD88/NF-κβ signaling pathways and its extract metabolite profile
Source: PLoS One. 2025 Apr 16;20(4):e0321018. doi: 10.1371/journal.pone.0321018 (PMC12002509; doi:10.1371/journal.pone.0321018)
Supplement: S1 Data — (DOCX) [file pone.0321018.s001.docx]

**The groups on the all the WB plates were placed according to the following sequence**

| **Groups** |
| --- |
| **Normal** |
| **Control (EtOH) group** |
| **Omeprazole** |
| **25 mg/kg PM-EtOH** |
| **50 mg/kg PM-EtOH** |
| **100 mg/kg PM-EtOH** |


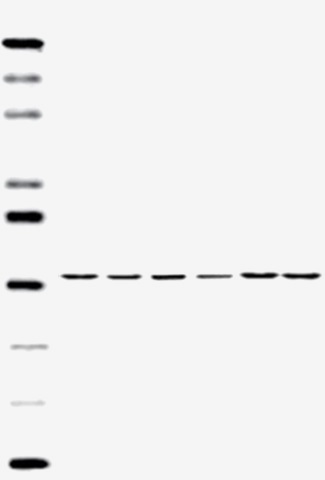


**β-actin (43 kDa)**


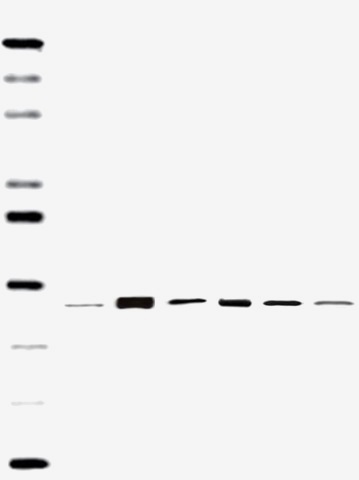


**HMGB1 (35 kDa)**


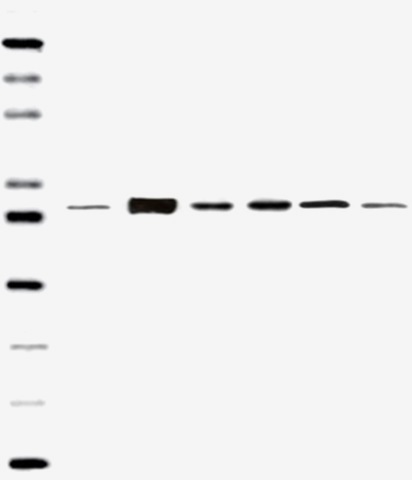


**NF-κB (65 kDa)**
