# Supplementary material for: In vivo anti-ulceration effect of Pancratium maritimum extract against ethanol-induced rats via NLRP3 inflammasome and HMGB1/TLR4/MYD88/NF-κβ signaling pathways and its extract metabolite profile
Source: PLoS One. 2025 Apr 16;20(4):e0321018. doi: 10.1371/journal.pone.0321018 (PMC12002509; doi:10.1371/journal.pone.0321018)
Supplement: S1 Table — (DOCX) [file pone.0321018.s002.docx]

**Table S1**. Gastric ulcer scoring system based on the severity of the ulcer.

| **Ulcer score (degree)** | **Gastric Lesion numbers** |
| --- | --- |
| 0 | No lesion |
| 1 | Mucosal edema and petechiae |
| 2 | One to five small lesions (1–2 mm), |
| 3 | More than five small lesions or one intermediate lesion (3–4 mm) |
| 4 | Two to more intermediate lesions or one gross lesion (>4 mm) |
| 5 | Perforated ulcers |
