# Supplementary figures and images for: In vivo anti-ulceration effect of Pancratium maritimum extract against ethanol-induced rats via NLRP3 inflammasome and HMGB1/TLR4/MYD88/NF-κβ signaling pathways and its extract metabolite profile
Source: PLoS One. 2025 Apr 16;20(4):e0321018. doi: 10.1371/journal.pone.0321018 (PMC12002509; doi:10.1371/journal.pone.0321018)

[*m/z* 120 [M+H-H2O-CO]

[*m/z* 149[M+H-NH2]


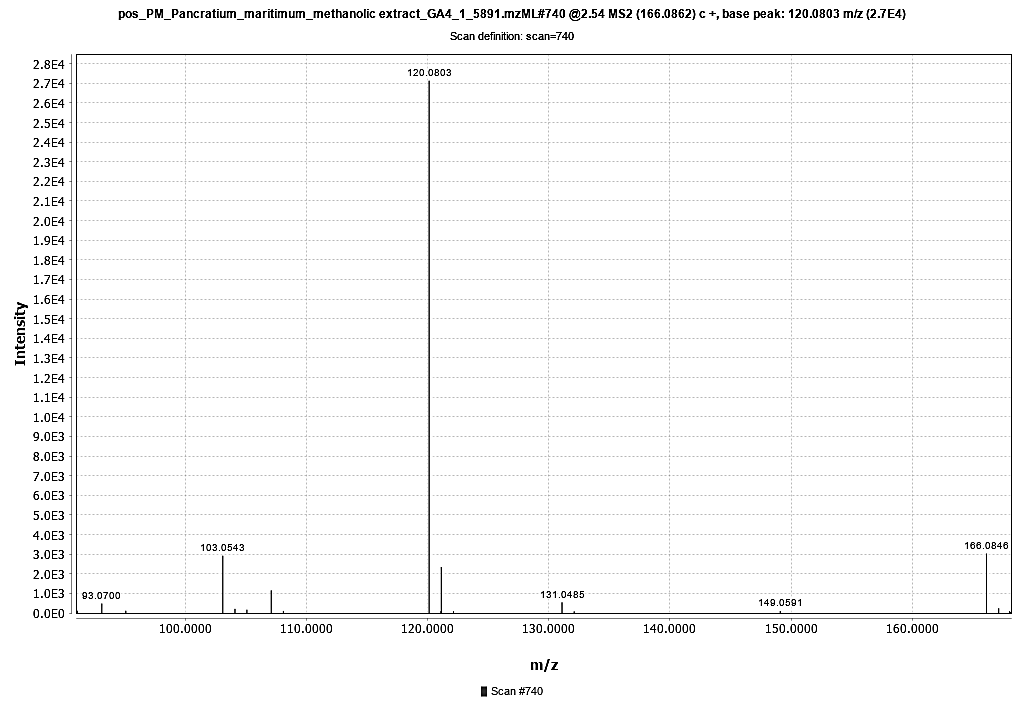


**Figure S1**: MS/MS fragmentation pattern of phenylalanine

Supplement: S1 Fig — (DOCX) [file pone.0321018.s003.docx]

**
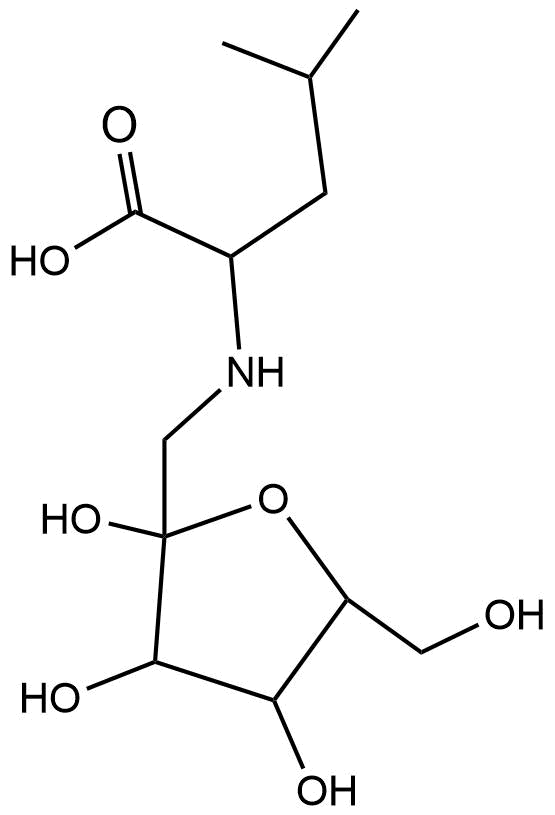

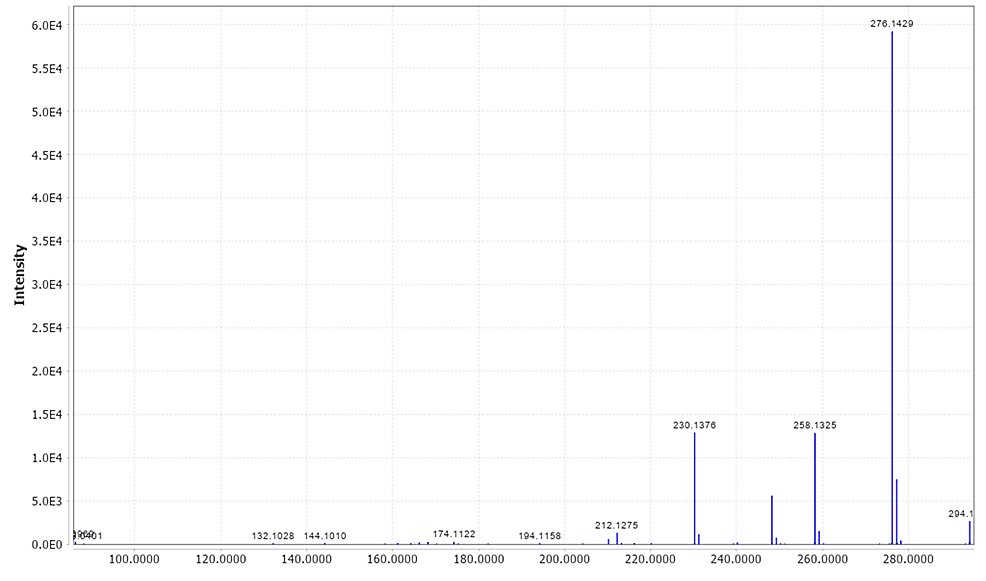
**

[M-H-H2O]

**Figure S2**: MS/MS fragmentation pattern of deoxy fructosyl leucine

[M-H-2H2O]

Supplement: S2 Fig — (DOCX) [file pone.0321018.s004.docx]

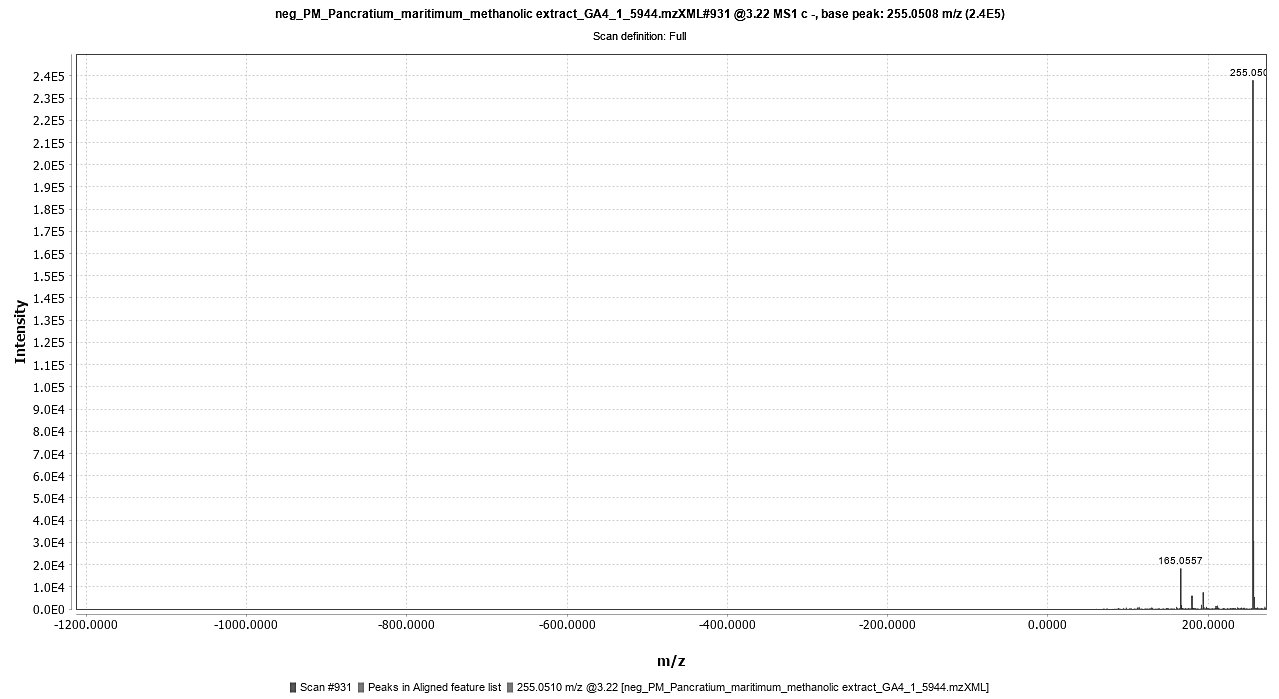


*m/z* 193 [M-H-CHO2-OH]-

*m/z* 165 [M-H-C2H2O3-OH]-

193

**Figure S3:** MS/MS fragmentation pattern of piscidic acid

Supplement: S3 Fig — (DOCX) [file pone.0321018.s005.docx]

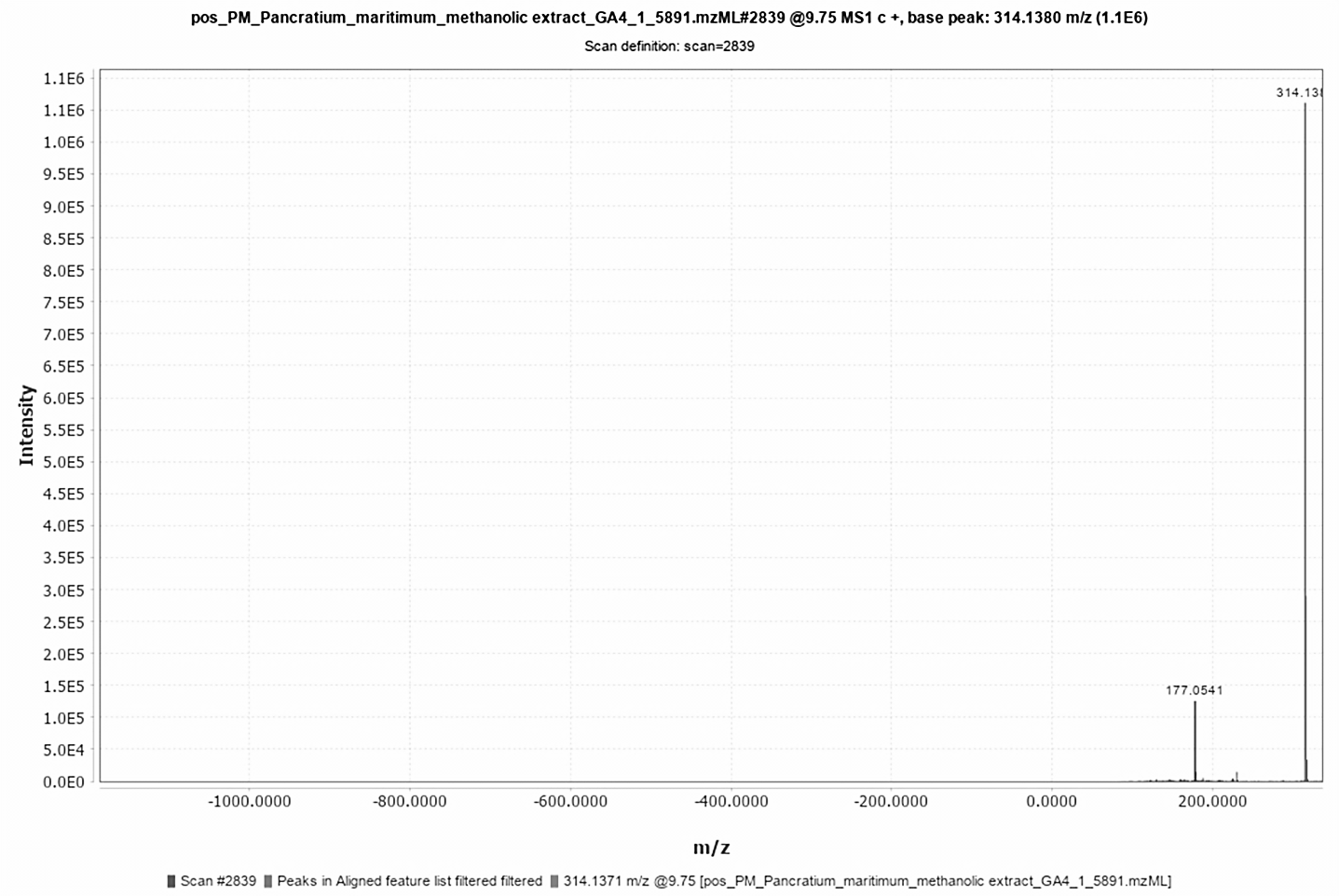


**
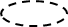
**

**Figure S4**: MS/MS fragmentation pattern of ferulyl tyramine

Supplement: S4 Fig — (DOCX) [file pone.0321018.s006.docx]

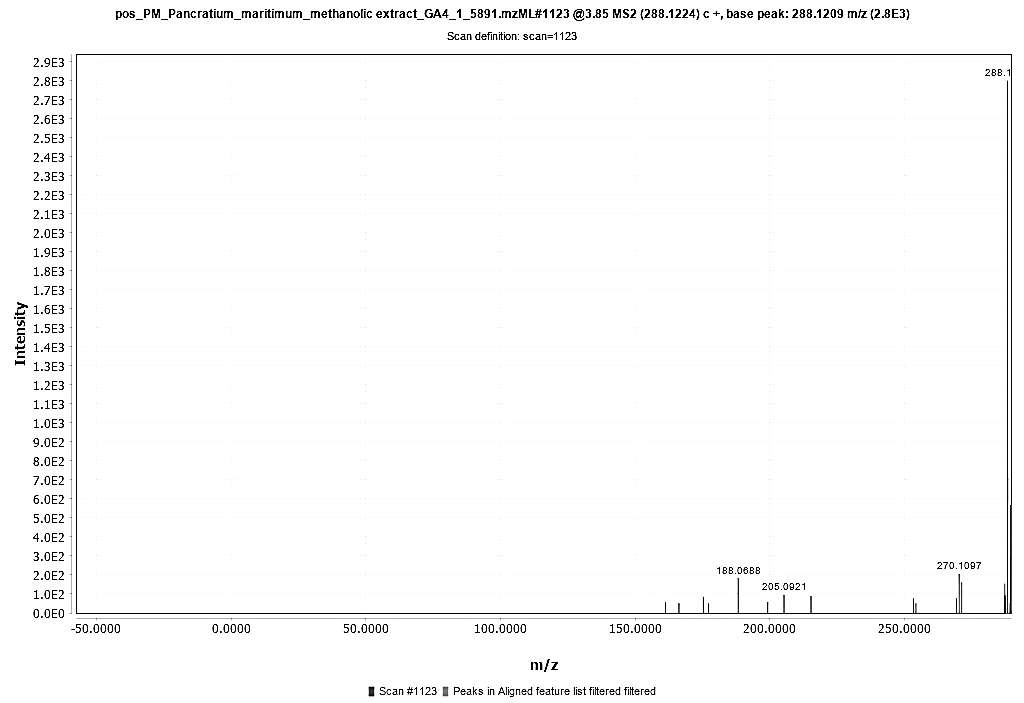


[*m/z* 177 [M-H-C6H9NO]

[*m/z* 270 [M-H-H2O]

**Figure S5.** MS/MS fragmentation pattern of lycorine

177

Supplement: S5 Fig — (DOCX) [file pone.0321018.s007.docx]

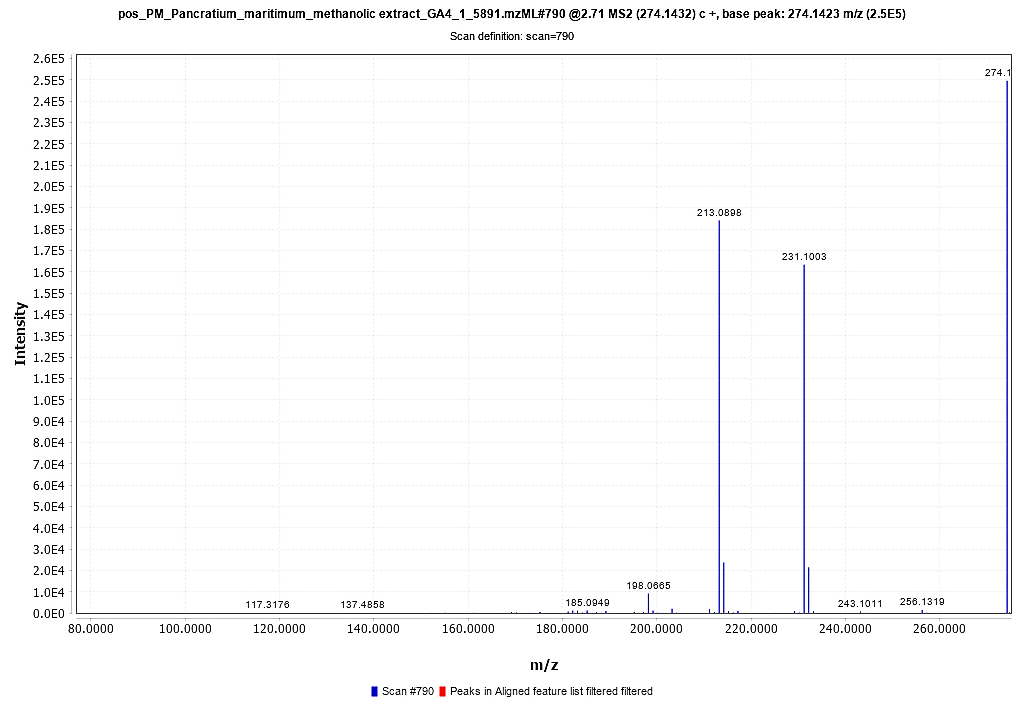


[213 *m/z* [M-H-C2H5N-H2O]

[231 *m/z* [M-H-C2H5N]

**Figure S6.** MS/MS fragmentation pattern of norgalanthamine

Supplement: S6 Fig — (DOCX) [file pone.0321018.s008.docx]

**
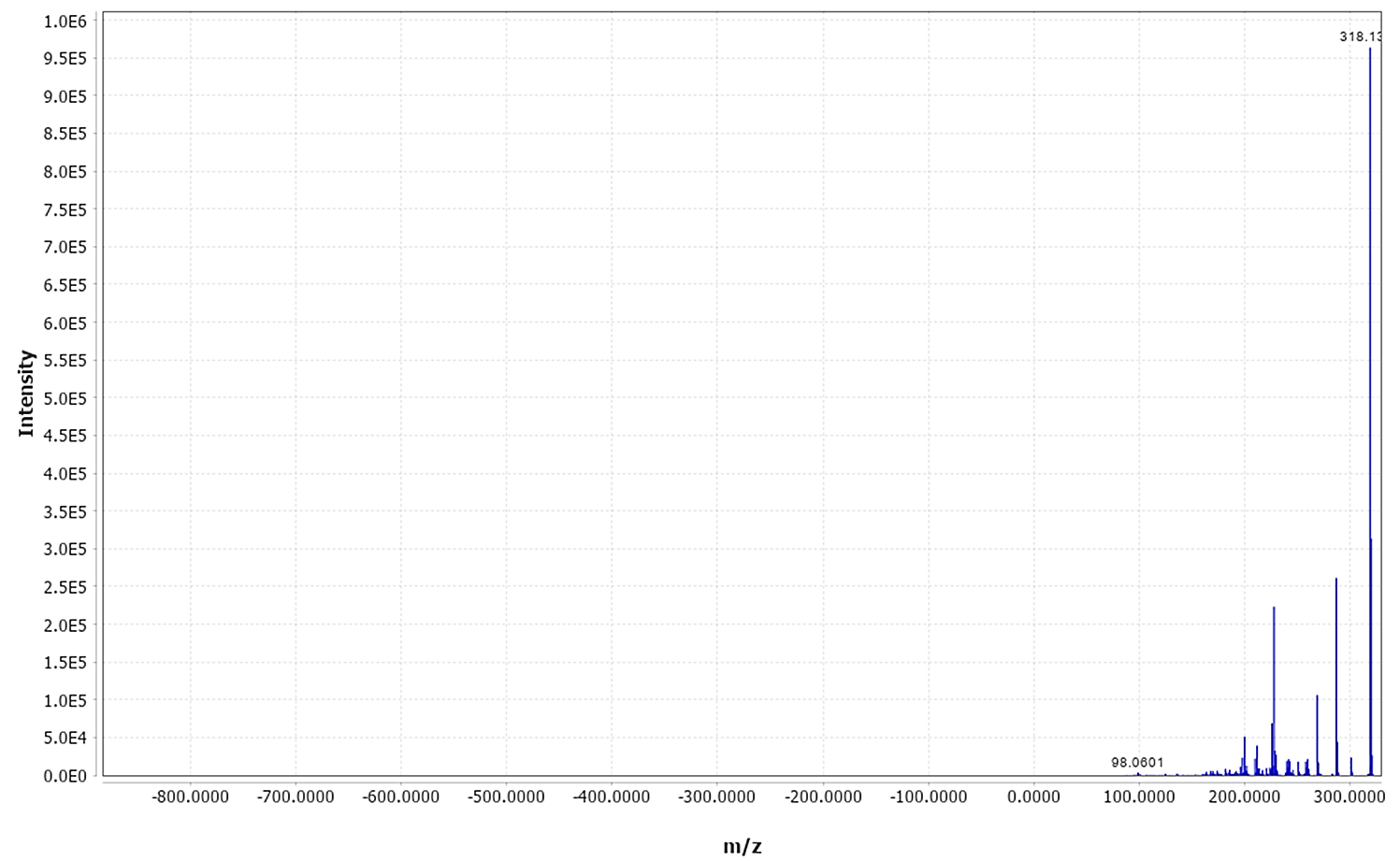
**

*m/z* 268 [M + H-CH3OH-H2O]

*m/z* 227 [M + H-CH3OH-H2O-C2H3N]

*m/z* 286 [M + H-CH3OH]

**Figure S7.** MS/MS fragmentation pattern of haemnathidine

Supplement: S7 Fig — (DOCX) [file pone.0321018.s009.docx]

**
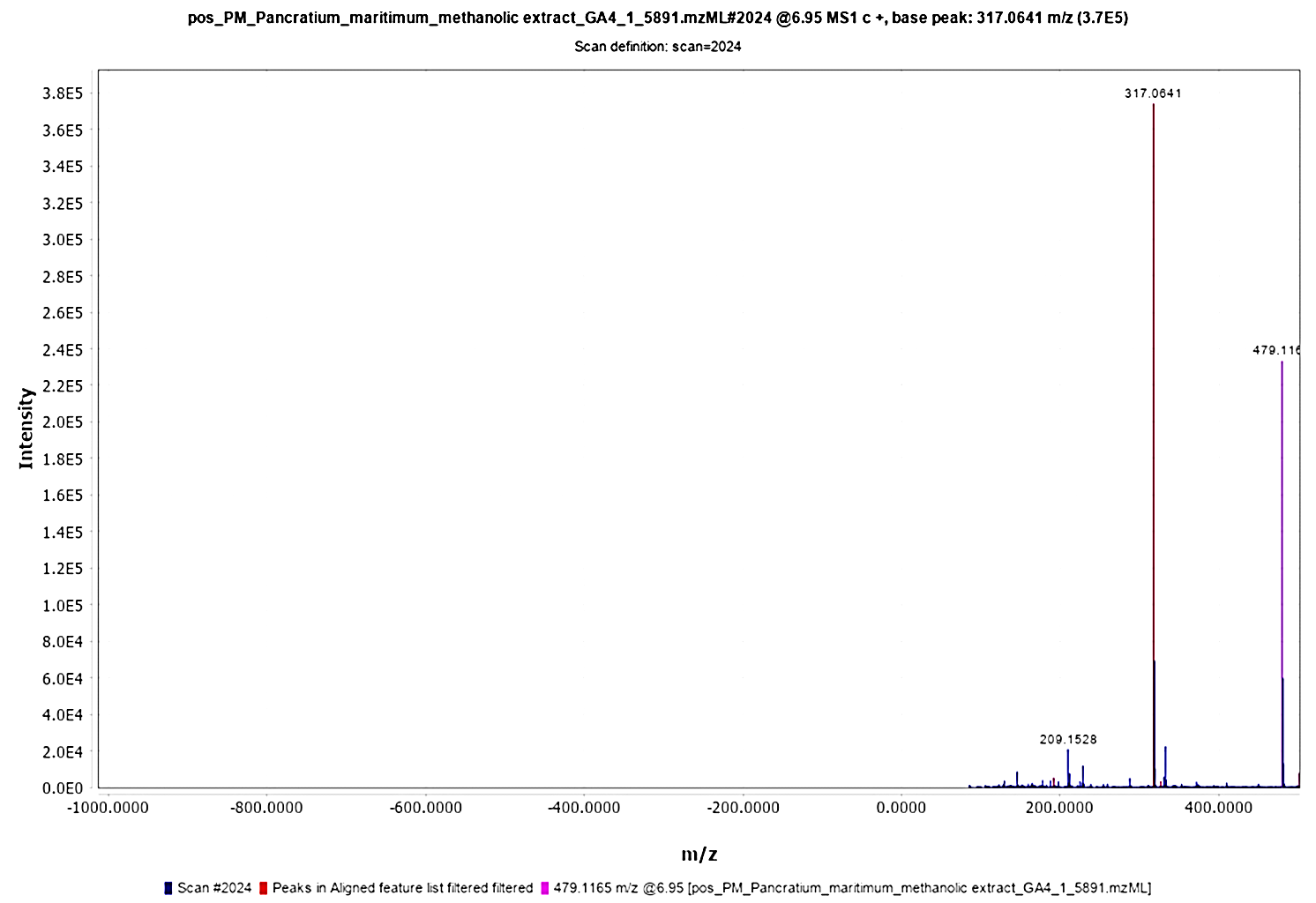
**

**
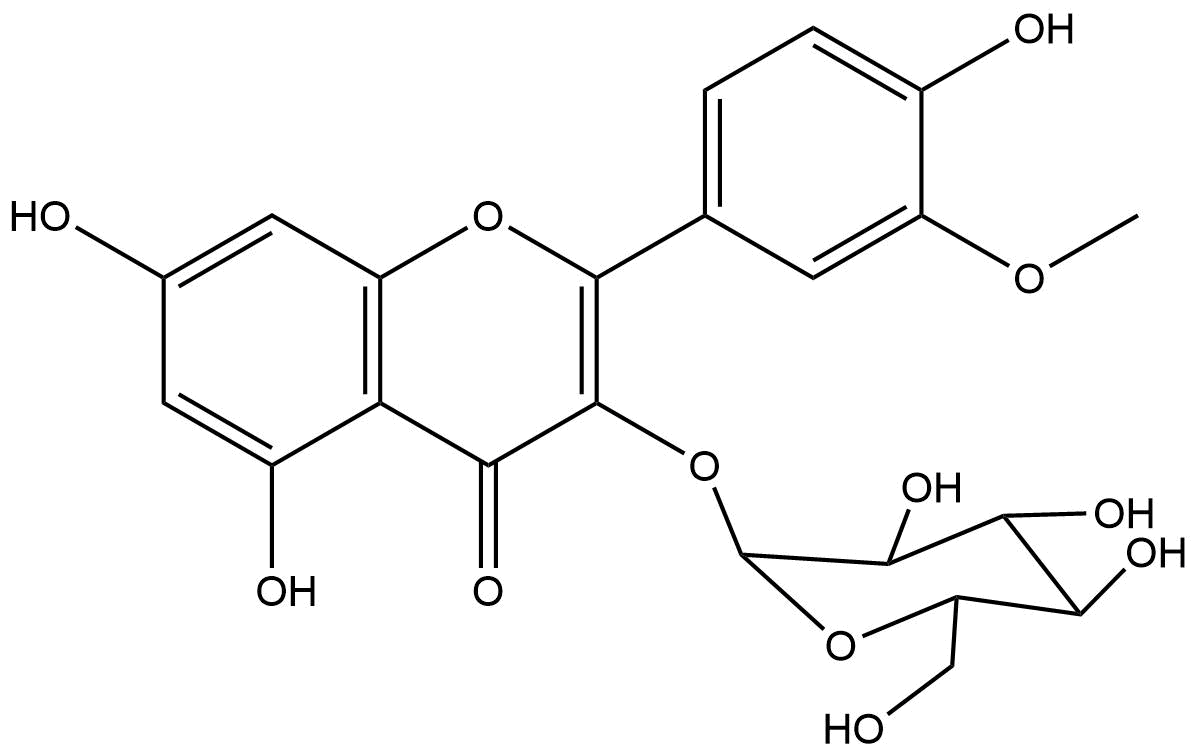
**

*m/z* 317

**Figure S8**: MS/MS fragmentation pattern of isorhamnetin-*O*-hexoside

Supplement: S8 Fig — (DOCX) [file pone.0321018.s010.docx]

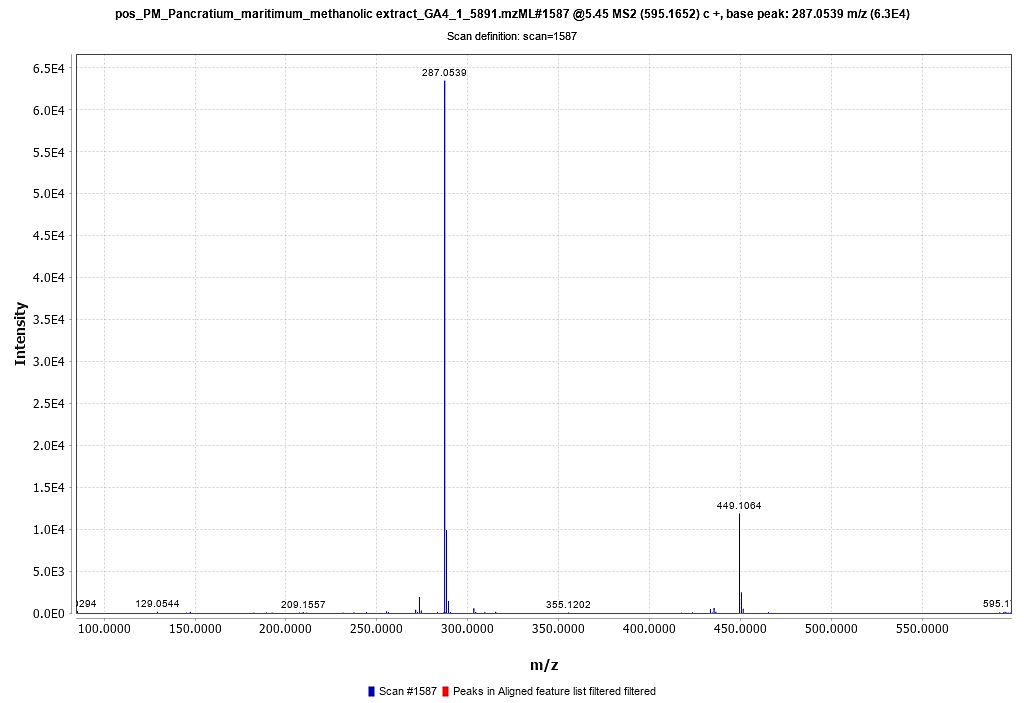

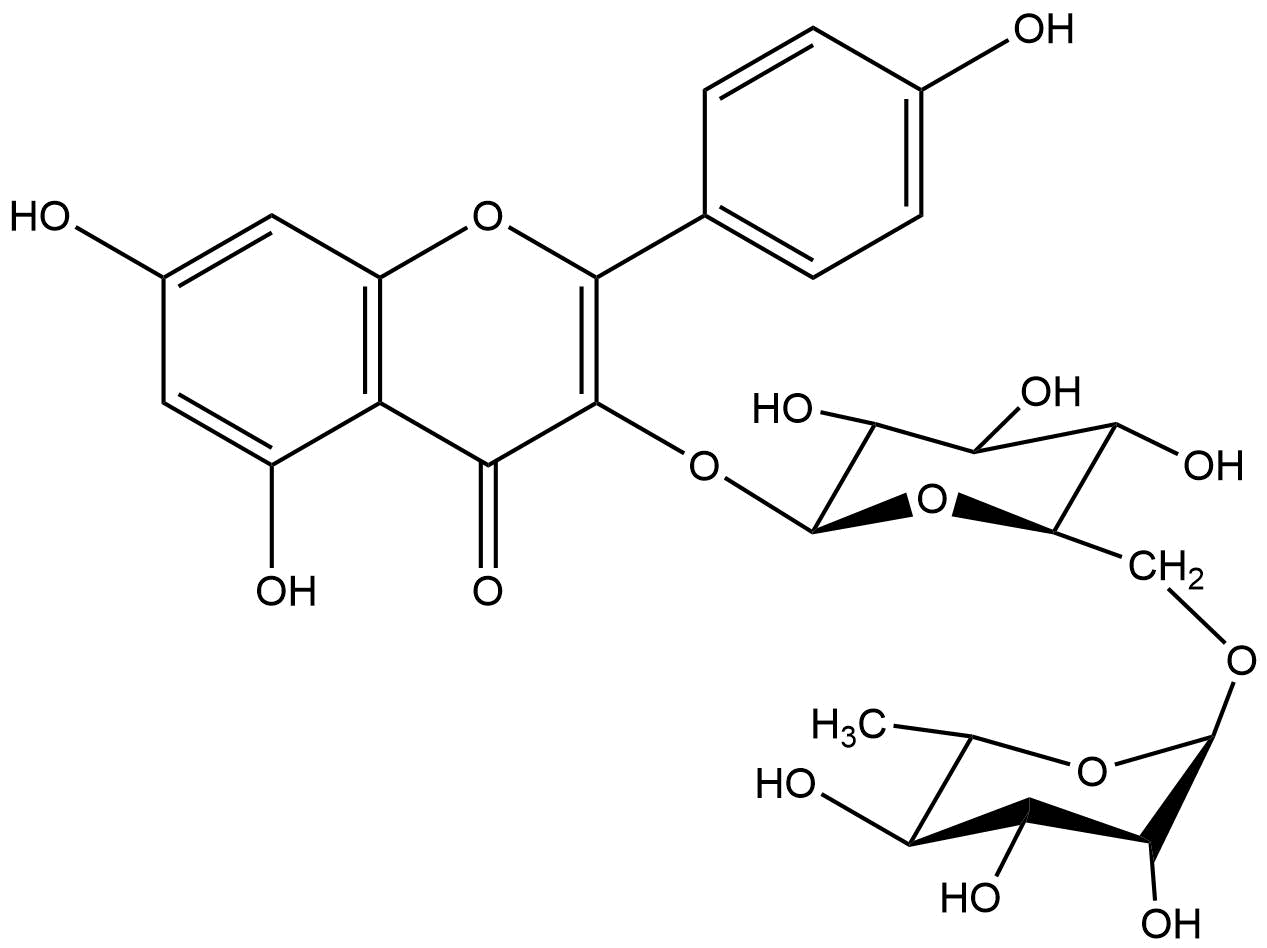


*m/z* 287

**Figure S9**: MS/MS fragmentation pattern of kaempferol-*O*-rutinoside

Supplement: S9 Fig — (DOCX) [file pone.0321018.s011.docx]

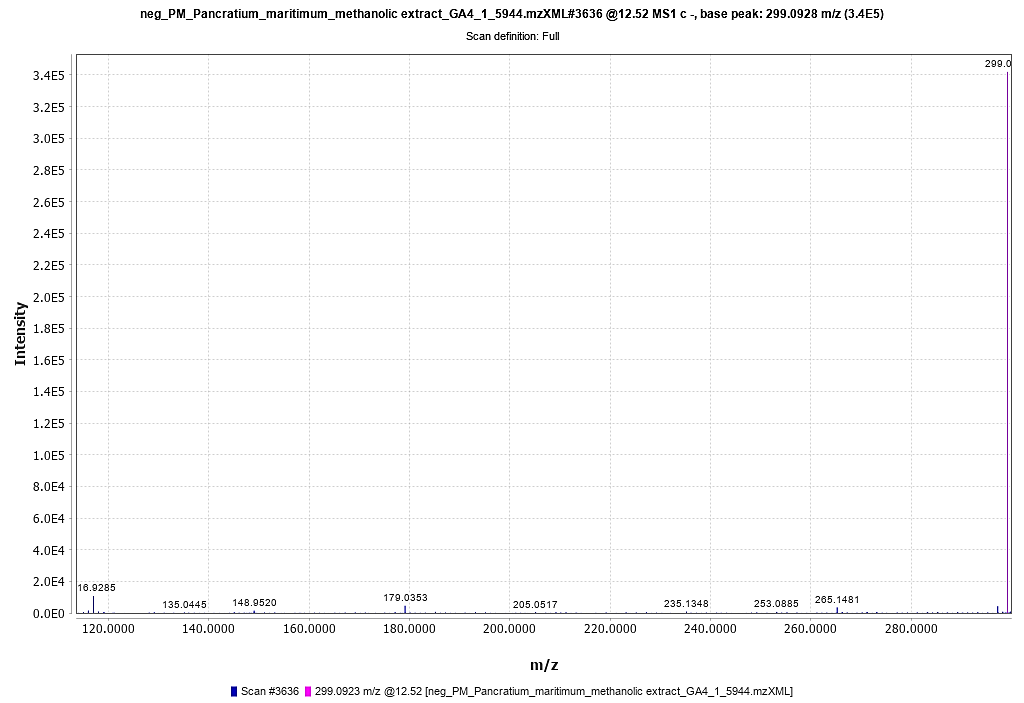


*m/z* 205

*m/z* 179

**Figure S10**: MS/MS fragmentation pattern of farrerol

Supplement: S10 Fig — (DOCX) [file pone.0321018.s012.docx]

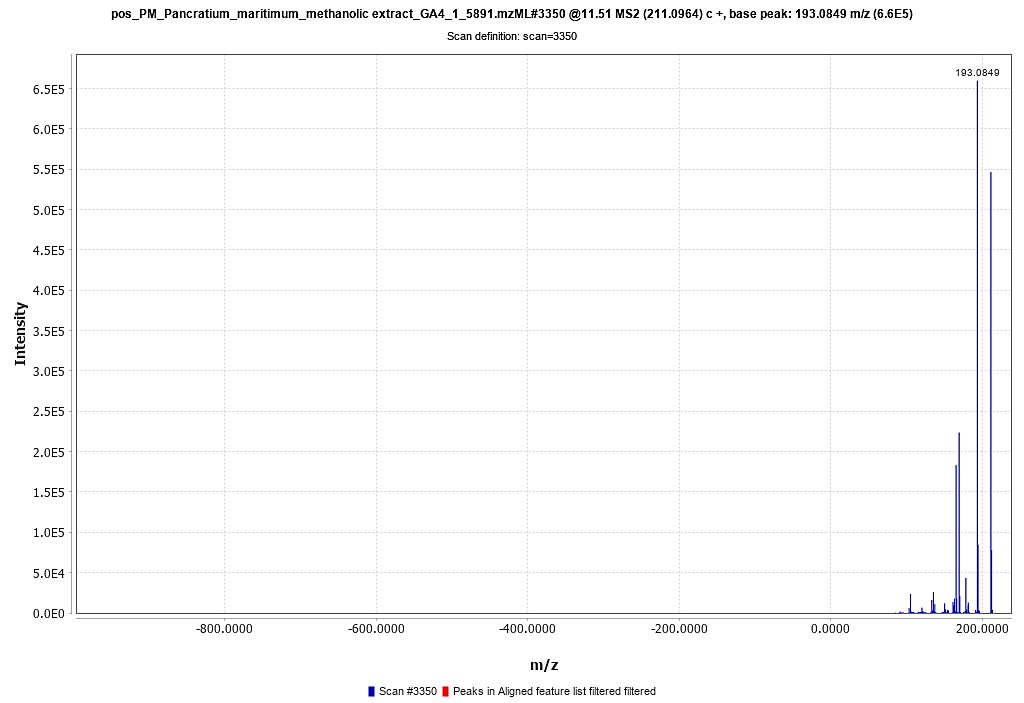


**Figure S11**: MS/MS fragmentation pattern of trimethoxy acetophenone

Supplement: S11 Fig — (DOCX) [file pone.0321018.s013.docx]
